# Characterization of paralogous *uncx* transcription factor encoding genes in zebrafish

**DOI:** 10.1016/j.gene.2019.100011

**Published:** 2019-03-08

**Authors:** Valeria Nittoli, Antonio Emidio Fortunato, Giulia Fasano, Ugo Coppola, Alessandra Gentile, Sylvie Maiella, Fernanda Langellotto, Immacolata Porreca, Raffaella De Paolo, Rita Marino, Marcella Fiengo, Aldo Donizetti, Francesco Aniello, Takashi Kondo, Filomena Ristoratore, Lorella M.T. Canzoniero, Denis Duboule, Stephen W. Wilson, Paolo Sordino

**Affiliations:** a Biology and Evolution of Marine Organisms, Zoological Station Anton Dohrn, 80121 Naples, Italy; b Immunrise Technologies, 75005 Paris, France; c Department of Developmental Genetics, Max Planck Institute for Heart and Lung Research, 61231 Bad Nauheim, Germany; d Orphanet, French National Institute for Health and Medical Research, 75014 Paris, France; e Wyss Institute for Biologically Inspired Engineering at Harvard University, 02115 Boston, USA; f Human Genetics,Wellcome Sanger Institute, CB10 1SA Hinxton, UK; g Department of Biology, University of Naples Federico II, 80126 Naples, Italy; h Laboratory for Developmental Genetics, RIKEN Center for Integrative Medical Sciences, 230-0045 Yokohama, Japan; i Department of Science and Technology, University of Sannio, 82100 Benevento, Italy; j School of Life Sciences, Federal Institute of Technology, 1015 Lausanne, Switzerland; k University of Geneva, 1205 Geneva, Switzerland; l Department of Cell and Developmental Biology, University College London, WC1E6BT London, UK

**Keywords:** Ace, acerebellar, AP, antero-posterior, CAMP, conserved ancestral microsyntenic pairs, CaP, caudal primary motor neuron axons, Ce, cerebellum, CRM, *cis*-regulatory module, CNE, conserved non-coding elements, CS, Corpuscle of Stannius, cyc, cyclops, Di, diencephalon, Elfn1, Extracellular Leucine Rich Repeat And Fibronectin Type III Domain Containing 1, Ey, eye, FB, forebrain, FGF, fibroblast growth factor, Flh, floating head, fss, fused-somites, HB, hindbrain, HM, hybridization mix, hpf, hours post fertilization, Hy, hypothalamus, Mical, molecule interacting with CasL, MO, morpholino, No, notochord, OP, olfactory placode, OT, optic tectum, PA, pharyngeal arches, PSM, presomitic mesoderm, ptc, patched, SC, spinal cord, Shh, sonic hedgehog, smu, slow-muscle-omitted, So, somites, syu, sonic-you, Te, telencephalon, Th, thalamus, TSGD, teleost-specific genome duplication, VLP, ventro-lateral-posterior, WIHC, whole-mount immunohistochemistry, WISH, whole-mount *in situ* hybridization, YE, yolk extension, Yo, yolk, yot, you-too, Uncx, TSGD, Zebrafish, Synteny, Signaling pathway, Development

## Abstract

The paired-type homeodomain transcription factor Uncx is involved in multiple processes of embryogenesis in vertebrates. Reasoning that zebrafish genes *uncx4.1* and *uncx* are orthologs of mouse *Uncx*, we studied their genomic environment and developmental expression. Evolutionary analyses indicate the zebrafish *uncx* genes as being paralogs deriving from teleost-specific whole-genome duplication. Whole-mount *in situ* mRNA hybridization of *uncx* transcripts in zebrafish embryos reveals novel expression domains, confirms those previously known, and suggests sub-functionalization of paralogs. Using genetic mutants and pharmacological inhibitors, we investigate the role of signaling pathways on the expression of zebrafish *uncx* genes in developing somites. In identifying putative functional role(s) of zebrafish *uncx* genes, we hypothesized that they encode transcription factors that coordinate growth and innervation of somitic muscles.

## Highlights

• The *Uncx4.1* and *Uncx* genes derive from the teleost-specific whole-genome duplication.
• *Uncx* genes are expressed during embryogenesis in unique and overlapping domains.
• *Uncx* gene expression during somite differentiation is regulated by FGF signaling.
• Synteny and expression profiles correlate *Uncx* genes with axon guidance.

## 1 Introduction

The *Uncx* gene (also known as *Uncx4.1*, *Phd1* and *Chx4*) encodes a transcription factor containing a paired-type homeodomain homolog to *Caenorhabditis elegans* UNC-4 homeoprotein (Miller et al., 1992; Rovescalli et al., 1996). The nematode UNC-4 controls synaptic choices of specific motor neurons in the ventral nerve cord by modulating their sensitivity to diffusible Wnt ligands (White et al., 1992; Miller and Niemeyer, 1995; Schneider et al., 2012). In *C. elegans* and *Drosophila melanogaster*, UNC-4 orthologs form a repressor complex with UNC-37, homolog of Groucho/TLE transcriptional co-repressor (Pflugrad et al., 1997; Winnier et al., 1999; Giot et al., 2003; Von Stetina et al., 2007). Vertebrate *Uncx* genes are implicated in multiple processes of embryogenesis, as suggested by their expression in olfactory epithelium, telencephalon, mesencephalon, spinal cord, branchial arches, kidney, somites, and forelimb autopod (Saito et al., 1996; Neidhardt et al., 1997). Many mechanisms underlying the role of Uncx have been proposed, including cell adhesion, axon guidance, cell cycle control and differentiation processes in postmitotic stages (Mansouri et al., 2000; Bussen et al., 2004; Asbreuk et al., 2006; Sewell et al., 2009; Skuntz et al., 2009; Sammeta et al., 2010; Rabe et al., 2012).

In vertebrates, *Uncx* is transcribed in sclerotomal cells surrounding the notochord, suggesting a conserved role as determinant of axial skeleton morphogenesis (Neidhardt et al., 1997; Mansouri et al., 1997; Koudijs et al., 2008; Sánchez and Sánchez, 2013; Retnoaji et al., 2014). *Uncx* functions are perhaps best understood in amniotes. Loss-of-function studies in mice support a role in the condensation of mesenchymal cells of the lateral sclerotome and proper development of pedicles, transverse processes, and proximal rib derivatives. Moreover, disruption to the establishment of antero-posterior (AP)-somite polarity in *Uncx* mutant mice suggests that this gene is required for the maintenance of posterior somite characteristics (Leitges et al., 2000; Mansouri et al., 2000).

*Uncx* transcription in the presomitic mesoderm (PSM) depends on *Delta*-*like 1* (*Dll1*) and is independent from signals of the axial structures, such as notochord-floor plate complex, whereas further maintenance requires Uncx itself (Barrantes et al., 1999; Mansouri et al., 2000; Schrägle et al., 2004; Sewell et al., 2009). A central role in the repression of *Uncx* expression in the anterior somite is played by a complex regulatory network that involves the basic helix–loop–helix transcription factor Mesp2, its downstream co-repressor Ripply, the homeodomain transcription factor MEOX1, and a cross-negative regulation with the T-box protein Tbx18 (Takahashi et al., 2000, Takahashi et al., 2003, Takahashi et al., 2013; Nakajima et al., 2006; Farin et al., 2008; Skuntz et al., 2009; Yabe et al., 2016). Recently, cell type-specific epigenetic regulation of *Uncx* gene expression has been associated with axon guidance in *C. elegans* (Zheng et al., 2013) and with human leukemia (Daniele et al., 2017). It has been proposed that *Uncx* is implicated in cell cycle progression of neuronal progenitor cells, survival of olfactory epithelium and differentiation of dopaminergic neurons (Sammeta et al., 2010; Rabe et al., 2012).

Although many advances have been made in dissecting the biological significance for development and the mechanisms of action of vertebrate *Uncx*, other aspects, including molecular evolution and roles in axonal growth, remain poorly defined. To elucidate the cascade of events accomplished by the Uncx proteins the zebrafish (*Danio rerio*) could be an ideal model due to its amenability to embryological and genetic approaches. However, to date *Uncx* homologs in zebrafish have not been characterized in detail. In this study, we performed genome and gene expression analyses of the zebrafish genes *uncx4.1* and *uncx*, with a focus on somite formation and innervation. Taken together, our results provide insights into the potential role of zebrafish *uncx* genes in the formation of spatially distinct muscle progenitor domains and in axon pathfinding.

## 2 Materials and methods

### 2.1 Molecular evolution

The protein sequences used for the evolutionary analysis were retrieved from the NCBI and Ensembl databases. The *Homo sapiens* UNCX protein was the initial query sequence employed for tBlastn searches (Gertz et al., 2006) in invertebrate and vertebrate genomes, and reciprocal Blasts were carried out on each genome. ClustalW was used to align the proteins selected for phylogenetic analysis with default parameters (Thompson et al., 1994). The phylogenetic tree was built with the Maximum-Likelihood estimation (MLE) using MEGA6 with 1000 replicates; the LG substitution model, with 0.2 as proportion of invariable sites (I) and 4 as gamma distribution parameter (γ), was selected (Tamura et al., 2013). The graphical representation was created with Dendroscope (Huson and Scornavacca, 2012). The synteny analysis between human and zebrafish chromosomes was performed with “Sinteny Database” and a sliding window size of 50 genes (Catchen et al., 2009). The syntenic survey between human and the tunicate *Ciona robusta* was performed mapping manually the genes on the scaffolds/chromosomes in Ensembl and Genomicus databases. Introns were mapped by using available public resources and designed, with a color code representation, on the protein alignment obtained using ClustalW. The analysis of genomic conservation was performed on ten sequences, employing mVISTA computational tool (Ratnere and Dubchak, 2009). To identify conserved non-coding sequences by VISTA, we employed LAGAN (global pair-wise and multiple alignments of finished sequences) with the following parameters: minimum Conservation Width for non-coding sequences (40 bp), minimum conservation identity (50%), and minimum Y value (20%). To improve the comparison of distant homologs, the translated anchoring in LAGAN/Shuffle-LAGAN was used.

### 2.2 Zebrafish stocks and husbandry

Zebrafish of wild-type AB, *sonic*-*you* (*syu*^*tbx392*^), *cyclops* (*cyc*^*b16*^), *acerebellar* (*ace*^*ti282a*^), *smoothened* (*smu*^*b577*^), *you*-*too* (*yot*^*ty119*^), *floating head* (*flh*^*n1*^) and *fused*-*somites* (*fss*^*te314a*^) lines were raised and maintained at 28 °C under a reproduction regime (14 h light/10 h dark cycle) at UCL (UK). All embryos were collected after natural spawning and staged in somites (s) and hours post fertilization (hpf) according to Kimmel et al. (1995). Fertilized embryos were kept in Petri dishes containing E3 medium (5 mM NaCl, 0.17 mM KCl, 0.33 mM CaCl_2_, 0.33 mM MgSO_4_, 1 × 10–5% Methylene-blue). Ethical approval for zebrafish experiments was obtained from local review panels and from the Home Office UK under the Animal Scientific Procedures Act 1986.

### 2.3 Actin filament staining

Whole-mount phalloidin staining was performed as described (Whitfield et al., 1996). Embryos were fixed with 4% paraformaldehyde (PFA), followed by permeabilization in 2% Triton X-100/PBS for 1.5 h and incubation in 2.5 μg/ml fluorescein-labeled phalloidin (Sigma) in PBS for 2 h in the dark at 4 °C. Embryos were then rinsed overnight and mounted in 70% glycerol/30% PBS prior to proceed to image acquisition.

### 2.4 Cloning and probe synthesis

A neurula stage zebrafish cDNA library (kind gift of D. Grunwald) prepared in the λ ZAP II vector (Stratagene) was screened for homeobox-containing genes by PCR with a primer annealing to the cloning site of the plasmid vector and a degenerate primer annealing to a conserved homeobox region (TTGACCCKCCKGTTYTGRAACCA). We cloned a 220 bp cDNA fragment of *uncx4.1* that was used as probe to screen 1.2/10^6^ recombinant plaques of the same library at moderate stringency. From a fourth screen, a Bluescript phagemid was rescued and its 2 kb insert sequenced, which encoded a full-length Uncx4.1 protein as judged by a BLAST search of GenBank and EMBL databases. To make riboprobes for WISH analysis, a 950 bp *uncx4.1*-containing pBluescript SK+ plasmid was linearized with *Eco*RI and transcribed with T7 for the antisense probe, or linearized with *Apa*I and transcribed using T3 polymerase for the sense riboprobe. A 562 bp cDNA fragment of the *uncx* gene coding sequence was amplified (Fwd: 5′-AGCCACCATCATGTGTACGA-3′ and Rev: 5′-CGGGAAGGAGTTTGTTTTGA-3′), cloned into pCR™ II-TOPO® vector following TOPO TA Cloning instruction manual, and sequenced. The TOPO TA plasmid was linearized with *Hin*dIII and transcribed using T7 polymerase for the antisense probe or linearized with ApaI and transcribed using SP6 polymerase for the sense probe (Suppl. Fig. 1).

### 2.5 Whole-mount *in situ* hybridization (WISH)

Zebrafish embryos (*n* = 20/group) at different stages of development were anaesthetized with tricaine MS-222, fixed by immersion in 4% PFA overnight at 4 °C, and eventually de-pigmented using 3% hydrogen peroxide and 1% KOH. Fixed embryos were stored in 100% methanol at −20 °C. Embryos were permeabilized by proteinase K treatment (10 μg/ml). The hybridization was carried out at 65 °C with the specific digoxigenin-labeled probes diluted in hybridization mix (HM: 50% formamide, 1.3× SSC, 5 mM EDTA, 50 μg/ml yeast RNA, 0.2% Tween 20, 0.5% CHAPS, 100 μg/ml heparin). Embryos were incubated with anti-digoxigenin alkaline phosphate-conjugated antibodies (1:5000; Roche) at 4 °C. Embryos were stained in BM Purple solution (Roche). Additionally, to detect mRNA of other markers (*shha*, *her1*, *mespaa*, *myod1*, *egr2b*), embryos were incubated in pre-staining buffer (100 mM Tris-HCl, 0.1% Tween 20) for 30 min and stained in Fast Red solution (Roche). After stopping the reaction, embryos were post-fixed in 4% paraformaldehyde in 1× phosphate-buffered saline (PBS) for 20 min and finally stored in 95% glycerol at 4 °C. Embryos were imaged using a Zeiss Axio Imager M1 microscope equipped with Axiocam digital camera (Zeiss). WISH experiments were performed in biological triplicates. No hybridization signal was detected using a sense probe on all developmental stages analyzed.

### 2.6 Whole-mount immunohistochemistry (WIHC)

Zebrafish embryos were collected and fixed as described for WISH. Embryos were permeabilized by proteinase K treatment (10 μg/ml), incubated with a blocking solution (NGS 3%) for 2 h and incubated with monoclonal primary antibodies (acetylated α-tubulin, 1:1000 (Sigma-Aldrich); znp1, 1:100 (DSHB); MF20, 1:10 (DSHB); S58, 1:10 (DSHB); F59, 1:10 (DSHB)) diluted in PBT containing 3% NGS. After several washes in PBT, embryos were incubated with biotinylated anti-mouse IgG (1:200) or IgA-FITCH (1:200) for 2 h. For chromogenic staining, embryos were incubated with avidine-biotine solution (Vectastain ABC kit, Vector Labs) and, then, with chromogenic substrate 3,3′–diaminobenzidine (DAB) until staining was sufficiently developed. For combined WISH-WIHC experiments, WIHC (znp1 or acetylated α-tubulin) was performed subsequently to WISH for *uncx4.1* mRNA. Embryos were imaged as described for WISH. WIHC and WISH-WIHC experiments were performed in biological triplicates.

### 2.7 Microinjections

mRNA: To generate synthetic mRNA for injection, the entire *uncx4.1* reading frame (ORF) was cloned into the vector pβUT2 which was made by cloning 5′ and 3′ UTRs of *Xenopus* β-globin (from pSP64T; Krieg and Melton, 1984) at either side of pBlueScript (Stratagene) with a synthetic polylinker replacing the *Bgl*II site of pSP64T. To clone the *uncx4.1* gene ORF in-frame with the Kozak consensus sequence, which increases the efficiency of translation initiation by ribosomes (Kozak, 1986), a PCR on the 2 kb *uncx4.1* insert (see Section 2.4) was performed using the following primers: GACGAAGGTACCCCACCATGATGGATAGCCGGATC and CCTATTGGTACCTCAGTGCATGTCTACATC. The 1340 bp PCR fragment was gel purified, cut with *Kpn*I (introduced into the sequence through the primers), and ligated into the *Kpn*I-digested pβUT2 plasmid. The DNA sequence of the insert was confirmed by sequencing. The plasmid was linearized with *Eco*RI and the gene was transcribed *in vitro* with the help of the T3 mMessage mMachine Kit (Ambion) yielding capped RNA for injection. The zebrafish full-length *shha*-containing pSP64T plasmid for mRNA injection was a kind gift of P. Ingham (Krauss et al., 1993). Synthetic capped *shha* and *uncx4.1* mRNAs were injected repeatedly (*n* > 3) at concentrations of 400, 200, and 200 pg per embryo, respectively. Injections were carried out on 1- to 2-cell stage embryos.

Morpholino: Gene knockdown was achieved by morpholino (MO) antisense oligonucleotides designed to disrupt splicing of pre-mRNA or inhibit translation of mRNA (Gene Tools). The amount and the sequence for various morpholinos used are as follows: 0.5 mM *uncx4.1*-atg-MO (blocking translation antisense morpholino), 5′-GATCCGGCTATCCATCATTGCATCT-3′; 0.5 mM *uncx4.1*-atg-mismatch-MO, 5′-GATgCGGgTATCCATCATaGCAaCT-3′; 0.8 mM *uncx*-atg-MO (blocking translation antisense morpholino) 5′-GATCCAGTATCCTGCTGTCCATCAT-3′; 0.5 and 0.8 mM ctrMO (standard control morpholino), 5′-CCTCTTACCTCAGTTACAATTTATA-3′. All MOs were injected into embryos at one to four cell stages. In total, we analyzed 254 embryos injected with the atg-MO against *uncx4.1*, and 294 embryos injected with atg-MO against *uncx*.

### 2.8 Pharmacological treatments

After partial dechorionation of zebrafish embryos (*n* = 20/group) at 6 hpf, the following chemical molecules were administered: 50 μM cyclopamine (Sigma-Aldrich), 50 μM SB431542 (Sigma-Aldrich), 40 μM DAPT (N-[N-(3,5-Difluorophenacetyl-L-alanyl)]-(*S*)-phenylglycine t-butyl ester; Calbiochem) and 20 μM SU5402 (Calbiochem). Embryos were kept in an incubator set to 28 °C for the duration of exposure until the desired developmental stage. Pharmacological treatments were performed in biological triplicates.

## 3 Results

### 3.1 Evolutionary analysis

**Fig. 1:**
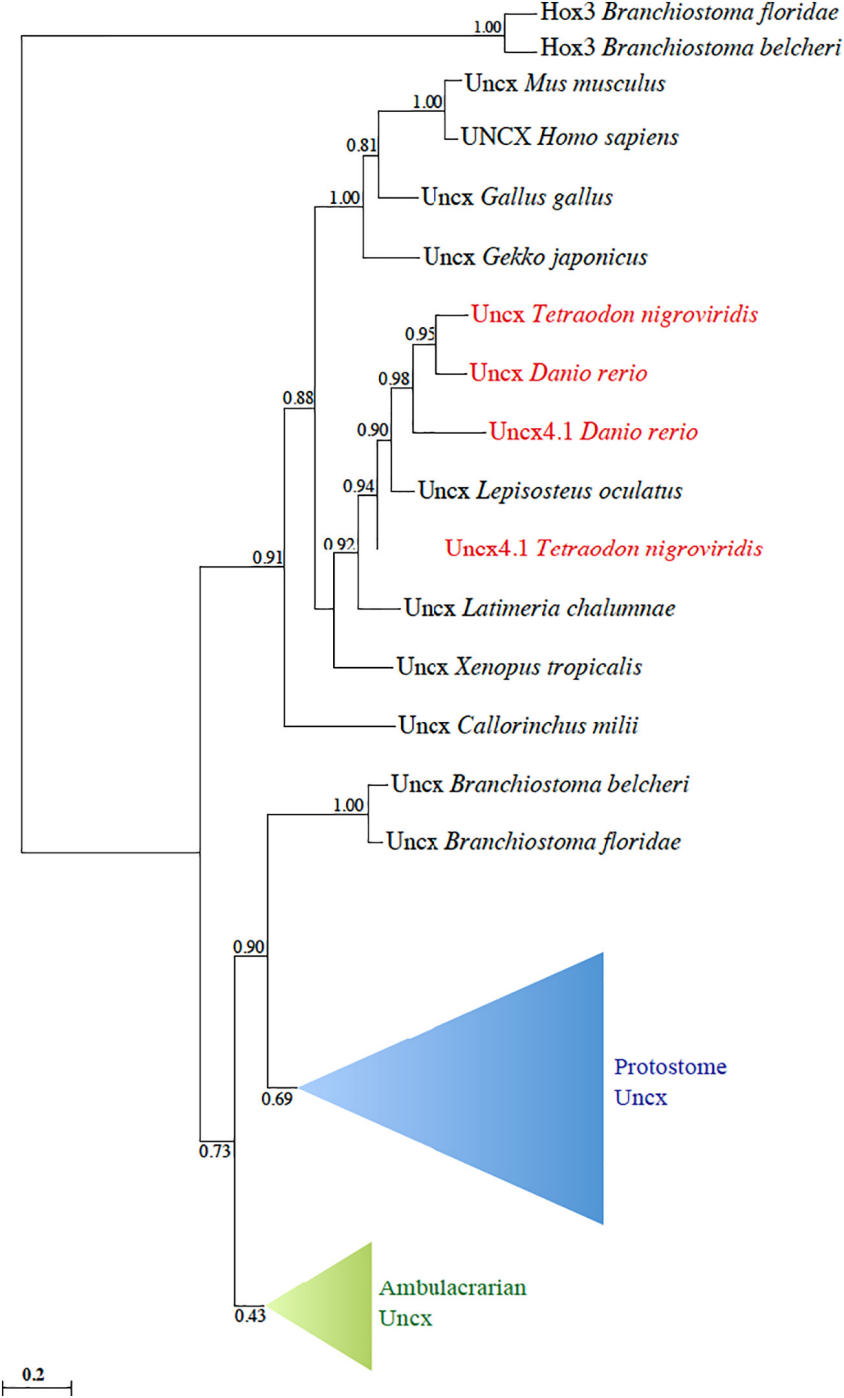
Phylogenetic analysis of Uncx proteins in metazoans. Numbers at branches represent replicates obtained using the Maximum Likelihood estimation method. The complete protein sequences were employed for tree inference. Uncx proteins deriving from teleost-specific genome duplication (TSGD) are shown in red. Protostome and Ambulacrarian Uncx proteins are grouped in the blue and green triangle, respectively. All sequences used in this analysis are reported in the Suppl. File 1. (For interpretation of the references to color in this figure legend, the reader is referred to the web version of this article.)

To decipher the evolutionary history of *Uncx* genes in metazoans, we performed a ML phylogenetic survey (Fig. 1) employing a collection of 25 manually curated protein sequences (Suppl. File 1) that encompasses: nematodes (*Caenorhabditis elegans*), mollusks (*Lottia gigantea*, *Crassostrea gigas*), annelids (*Capitella teleta*), brachiopods (*Lingula anatina*), hemichordates (*Saccoglossus kowalevskii*), echinoderms (*Strongylocentrotus purpuratus*, *Acanthaster planci*), cephalochordates (*Branchiostoma belcheri*, *Branchiostoma floridae*), and vertebrates (*Callorhinchus milii*, *Lepisosteus oculatus*, *Latimeria chalumnae*, *Danio rerio*, *Tetraodon nigroviridis*, *Xenopus tropicalis*, *Gallus gallus*, *Gekko japonicus*, *Mus musculus*, *Homo sapiens*). Selected outgroups were two cephalochordate Hox3 protein sequences from *B. belcheri* and *B. floridae*. We also found Uncx proteins in other genomes but these were excluded from the phylogeny due to their high molecular divergence (*e.g.*, *Ciona robusta* and *Takifugu rubripes*) or partial sequence (*e.g.*, *Nematostella vectensis*) (Suppl. File 2). Our genome search and phylogeny strongly indicated the existence of a single *Uncx* gene arisen at the root of bilaterians, as suggested by its presence in the cnidarian *Nematostella vectensis* genome (Ryan et al., 2006; Suppl. File 2). This gene has been affected by local duplications in invertebrates like *C. teleta*, *Drosophila melanogaster*, *S. kowalevskii*, and *Ciona robusta*, and lost in the placozoan *Trichoplax adhaerens*. Among vertebrates, we found a divergent Uncx protein in hagfish (*Eptatretus burgeri*), while no ortholog was identified in the lamprey genome (*Petromyzon marinus*) (Fig. 1; Suppl. File 2). Instead, a duplication event has been identified in teleosts (*D. rerio*, *T. nigroviridis*, *Takifugu rubripes*), possibly due to the Teleost-Specific Whole-Genome Duplication (TSGD) (Taylor et al., 2001; Taylor et al., 2003; Jaillon et al., 2004; Kuraku and Meyer, 2009). The analysis of gene structure unraveled the preservation of intron positions in *Uncx* genes, supporting their orthology from invertebrates to vertebrates (Suppl. File 3).

**Fig. 2:**
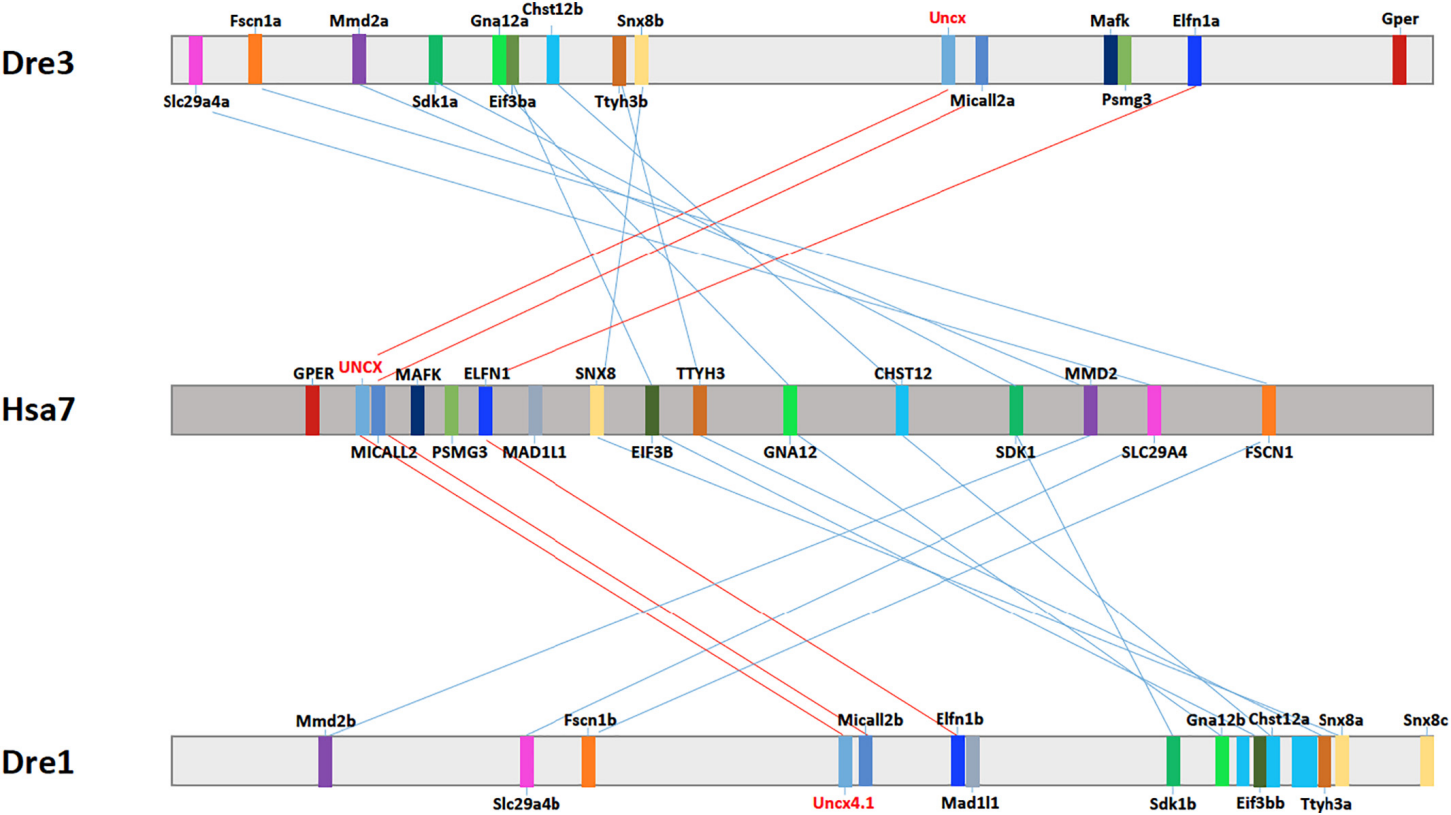
Synteny of *Uncx* genes in vertebrates. Horizontal bars represent orthologous genomic regions of human (*H. sapiens*, Hsa7) and zebrafish (*D. rerio*, Dre3 and Dre1). Orthologous genes are shown with same colors and are connected by lines. Red lines highlight a conserved microsynteny involving *Uncx*, *Micall2* and *Elfn1* genes. (For interpretation of the references to color in this figure legend, the reader is referred to the web version of this article.)

The study of the *Uncx* genomic *locus* revealed a high degree of synteny between tetrapods as human (Chr7) and teleosts as zebrafish (Chr1, Chr3), showing the preservation of 11 genes close to *Uncx*: the conservation of this cluster supports a TSGD-origin for zebrafish *Uncx* genes. The absence of some of these genes (*e.g.*, *gper*) in one of the two syntenic clusters suggests the secondary loss of TSGD-derived duplicates (Fig. 2).

Our survey also expanded our understanding of conservation of the *Uncx* genomic locus (Woolfe and Elgar, 2007), demonstrating high synteny in gnathostomes. In particular, we uncovered the presence of two conserved microsyntenic clusters. First, a gene triplet composed of *Uncx*, *Micall2* and *Elfn1* genes is present in gnathostomes. Mical (molecule interacting with CasL) and Micall are cytosolic multidomain proteins that have been associated to axon guidance, cell movement, cell-cell junction formation, vesicle trafficking, and cancer cell metastasis (Xue et al., 2010). Elfn1 (Extracellular Leucine Rich Repeat And Fibronectin Type III Domain Containing 1) is a protein specifically present in excitatory synapses, where it acts as a regulator of presynaptic release probably to direct interneuron recruitment (Cao et al., 2015). Despite the absence of synteny between Olfactores (Tunicata and Vertebrata) and other Metazoans, we traced back a gene duplet formed by *Uncx* and *Elfn1* genes in *C. robusta* (Chr11) and *H. sapiens* (Chr7) genomes (Suppl. Fig. 2). Importantly, this gene pair has been retained in all gnathostomes (data not shown). Concerning invertebrates, we also found two *Uncx* genes on the same chromosomal region in *D. melanogaster*, *C. teleta*, and *S. kowalevskii* (Suppl. Fig. 3). This *Uncx* duplet is flanked by *Alx*, which encodes a transcription factor with chondrogenic and other functions in vertebrates (Gordon et al., 1996), whereas *Alx* has been lost in *Drosophila* (Ryan et al., 2006). Furthermore, the amphioxus *B. floridae* ortholog clustered with the homeobox *Rx* gene (Irimia et al., 2012), essential for eye development (Sinn and Wittbrodt, 2013) (Suppl. Fig. 3).

Next, we sought to study the genomic region between the *Uncx* and *Micall2* genes, a duplet present only in gnathostomes and that has undergone duplication in teleosts (*uncx4.1*-*uncx*, *micall2a*-*micall2b*) (Fig. 2). A VISTA analysis revealed some conserved peaks within this gene duplet (Suppl. Fig. 4). Then, we analyzed the genomic *locus* of *Uncx* in ten metazoans selecting *ca.* 3000 base pairs downstream and upstream of the *Uncx* orthologous genes (Suppl. Fig. 5). Our plot revealed a conserved upstream region, whose traces are visible also in *C. gigas*, in *L. anatina* and *C. robusta*. With respect to vertebrates, teleosts did not show plain conservation in the upstream region (orange box) and in the second intron (green box). Notably, the upstream peak pattern differs between teleost paralogs (blue box). Altogether, this in-depth study of *Uncx* evolution defines this gene as an ultra-conserved homeobox gene with a complex evolutionary scenario in vertebrates.

### 3.2 Uncx expression during embryonic development

To generate probes for mRNA *in situ* hybridization, we first cloned the full-length transcripts of *uncx4.1* (NM_001020780) and *uncx* (XM_005164204.4). In order to have a spatio-temporal overview of *uncx* gene expression during zebrafish embryogenesis, we performed a whole-mount *in situ* hybridization (WISH) at various developmental stages until 48 hours post fertilization (hpf).

#### 3.2.1 Uncx4.1 (NM_001020780.2)

**Fig. 3:**
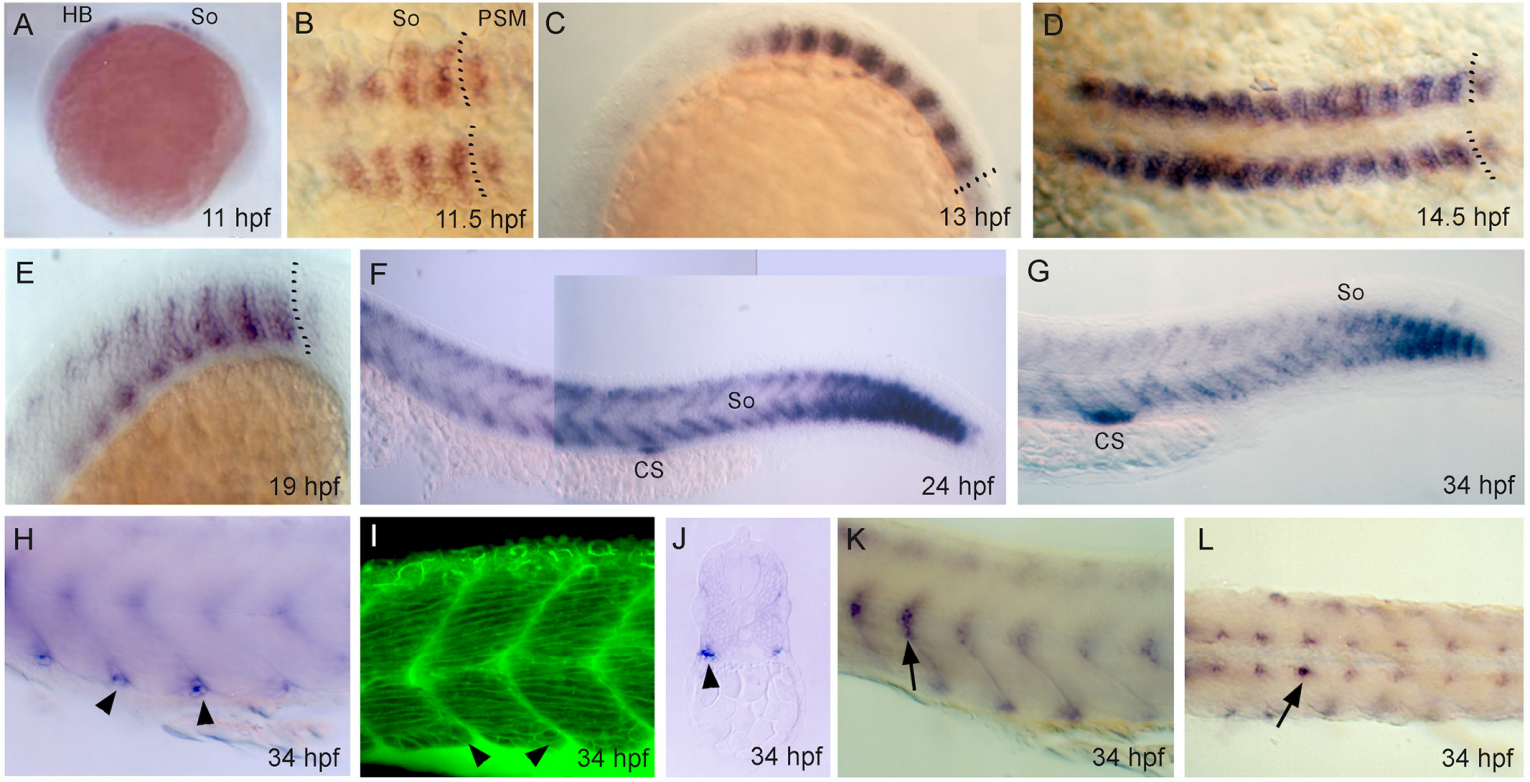
Expression of *uncx4.1* gene during somitogenesis. Whole-mount *in situ* hybridization of *uncx4.1* at (A) 11 hours post fertilization (hpf), (B) 11.5 hpf, (C) 13 hpf, (D) 14.5 hpf, (E) 19 hpf, (F) 24 hpf, and (G–L) 34 hpf. (A, C, E–H, J, K) Lateral view, (B, D) dorsal view, and (I) transversal section. (A–H, J–L) Anterior to left, and (I) toward viewer. (A) Expression in early somites (So) at 11 hpf. HB = hindbrain. (B–D) Expression in somites from 11.5 to 14.5 hpf. Dotted lines show boundary between presomitic mesoderm (PSM) and last formed somite. (E) Expression is lost in the myoseptum and is restricted posteriorly in developing somites at 10 hpf. (F) Expression is restricted to dorsal and ventral margins of anterior somites at 24 hpf, and (G) disappears dorsally at 34 hpf. CS = Corpuscle of Stannius, So = somites. (H) Arrowheads indicate *in situ* hybridization staining in ventro-latero-posterior cells (VLP) at 34 hpf. (I) Arrowheads indicate VLP cells in phalloidin-stained somites at 34 hpf. (J) Arrowhead indicates expression in VLP cells in transversal section at 16th somite level at 34 hpf. (K, L) Arrows indicate expression in sclerotome cells in (K) lateral and (L) dorsal view at 34 hpf.

Recently, tomographic data based on low-input RNA sequencing and mathematical image reconstruction have revealed early expression of *uncx4.1* in the shield at 5 hpf (Junker et al., 2014). In this study, the earliest evidence of *uncx4.1* expression was detected in all but the most anterior cells within each somite (11 hpf) (Fig. 3A). During early somitogenesis, *uncx4.1* expression is reiterated in newly forming metameric blocks (14.5 hpf) (Fig. 3B–D). Subsequently, expression disappears from the myoseptum to dorsal and ventral margins (19 hpf) (Fig. 3E) to become restricted in few boundary cells positioned in the ventral lateral posterior (VLP) tip of the somite (24–34 hpf) (Fig. 3F–H, J). VLP cells can be easily recognized at the cellular level with differential interference contrast optics, due to the round cell shape compared to the elongated fibroblasts. Phalloidin staining of 34 hpf embryos supports the view that *uncx4.1* transcripts mark cells of the future myomere that are morphologically distinct from the main adaxial somite portion (Fig. 3I). We also found *uncx4.1*-expressing cells on both sides of the notochord, perhaps corresponding to the fish sclerotome, the myogenic contribution to backbone formation (Morin-Kensicki and Eisen, 1997) (Fig. 3K, L).

**Fig. 4:**
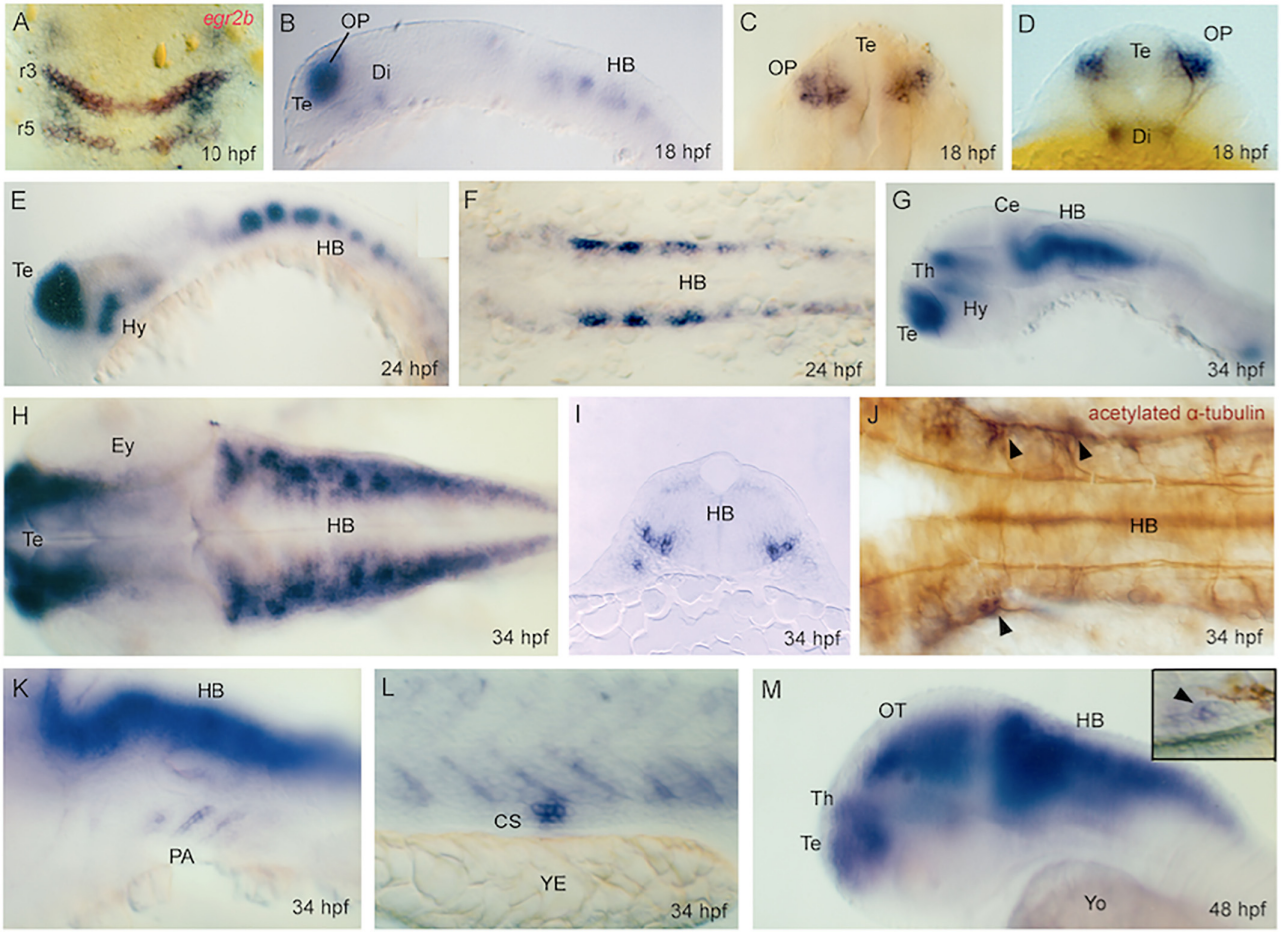
Non-somitic expression of *uncx4.1*. Whole-mount *in situ* hybridization of *uncx4.1* at (A) tailbud stage, (B–D) 18 hours post fertilization (hpf), (E, F) 24 hpf, (G–L) 34 hpf, and (M) 48 hpf. (A, C, F, H, J) Dorsal view, (B, E, G, H, K–M) lateral view, and (D, I) frontal view. (A, C) Anterior to top, (B, E–H, J–M) to left, and (D, I) toward viewer. (A) Early expression in rhombomeres 3–5 (r3, r5) as shown by double *in situ* labeling with *egr2b* riboprobe. Expression in telencephalon (Te), olfactory placodes (OP), hypothalamus (Hy), and prospective hindbrain (HB) at (B–D) 18 hpf and (E, F) 24 hpf. Di = diencephalon, HB = hindbrain, Hy = hypothalamus, OP = olfactory placode, Te = telencephalon. (G, H) Expression extends to thalamus at 34 hpf. Ce = cerebellum, Ey = eye, Th = thalamus. (I) Transversal section showing expression in neuronal progenitor cells in the hindbrain at 34 hpf. (J) Arrowheads indicate expression in the hindbrain near exit roots of branchial motor neurons as shown by double *in situ* labeling with acetylated α-tubulin antibody at 34 hpf. Expression (K) in pharyngeal arches and (L) Corpuscle of Stannius at 34 hpf in lateral view. CS = Corpuscle of Stannius, PA = pharyngeal arches, YE = yolk extension. (M) Expression in central nervous system and (inset) Corpuscle of Stannius at 48 hpf. OT = optic tectum, Yo = yolk.

During neurogenesis, *uncx4.1* is expressed in specific regions of the central nervous system (CNS). Early *uncx4.1* mRNA signal is found in rhombomeres 2–4 at 10 hpf (tailbud stage), as shown by double labeling with *egr2b*, a marker of early hindbrain rhombomeres 3 and 5 (Figs. 3A, 4A). During development, *uncx4.1* expression is visible in the olfactory placodes, telencephalon, and diencephalon, at 18–24 hpf (Fig. 4B–F), and in the ventral thalamus, pre-tectum, cerebellum, pharyngeal arches and pronephric ducts, at 34 hpf (Fig. 4G–L). The position of hindbrain cell bodies expressing *uncx4.1* coincided with the ventro-lateral exit roots of branchial motor neurons (Fig. 4H–J). At 48 hpf, we observed a widespread diffuse pattern for *uncx4.1* in the developing brain (Fig. 4M).

#### 3.2.2 Uncx (XM_005164204.4)

**Fig. 5:**
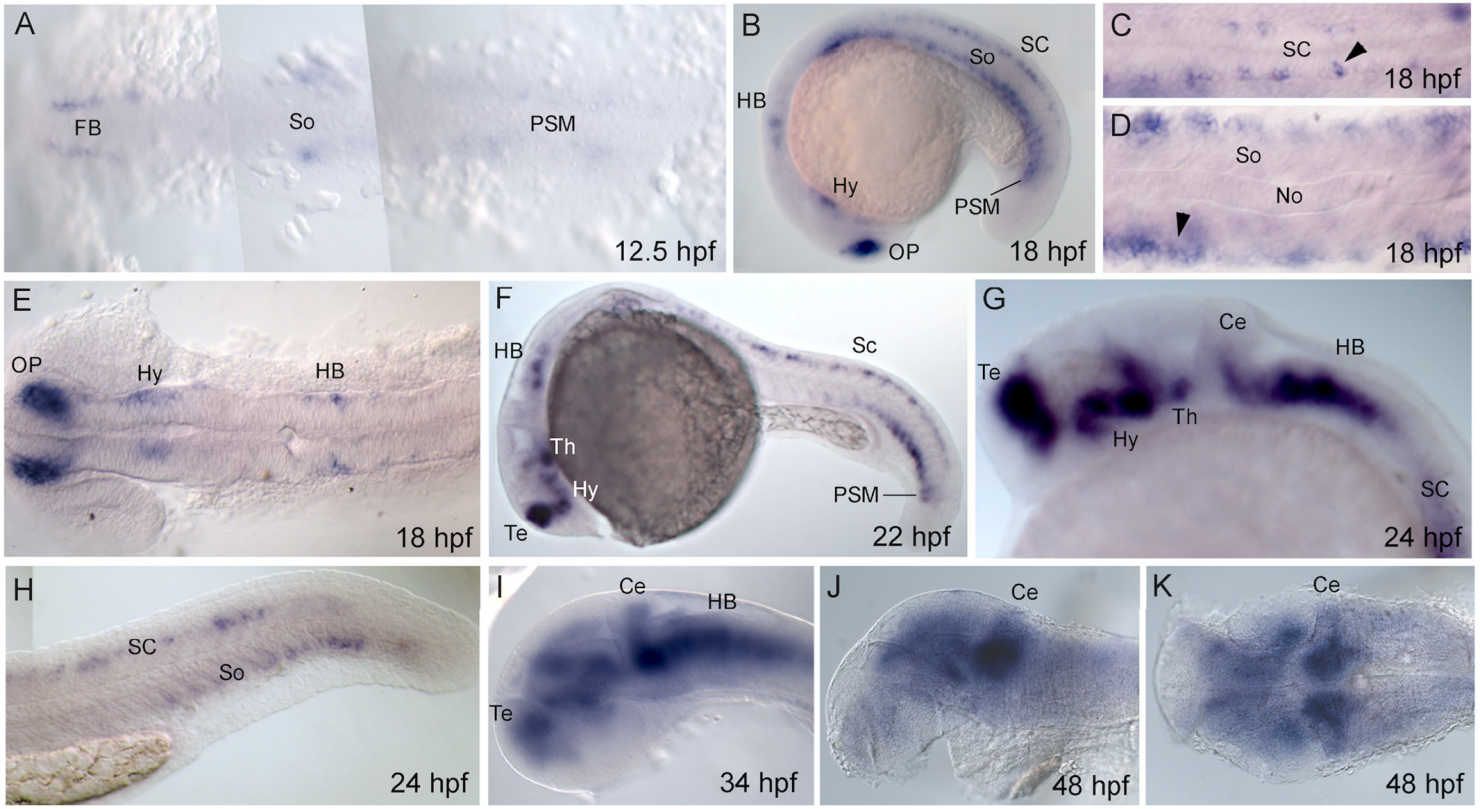
Expression of *uncx* during embryogenesis. Whole-mount *in situ* hybridization of *uncx* at (A) 12.5 hours post fertilization (hpf), (B–E) 18 hpf, (F) 22 hpf, (G, H) 24 hpf, (I) 34 hpf, and (J, K) 48 hpf. (A, C–E, K) Dorsal view, and (B, F–J) lateral view. Anterior to left. (A) Expression in prospective forebrain (FB) and hindbrain (HB) at 12.5 hpf. FB = forebrain, PSM = presomitic mesoderm, So = somites. (B–E) Expression of *uncx* at 18 hpf. HB = hindbrain, Hy = hypothalamus, No = notochord, OP = olfactory placode, SC = spinal cord, So = somites. (F) Expression at 22 hpf. Th = thalamus. (G, H) Expression at 24 hpf. Ce = cerebellum, Te = telencephalon. (I) Expression at 34 hpf. (J, K) Expression at 48 hpf.

Initially, weak signal of the *uncx* transcript is seen in bilateral columns of forebrain cell bodies, in the hindbrain, and in trunk mesodermal cells extending to PSM (12.5 hpf) (Fig. 5A). At 18 hpf, *uncx* expression is found in the olfactory placodes, telencephalon, diencephalon, hindbrain, spinal cord and ventral somites (Fig. 5B–E). At 22–24 hpf, expression is also present in pre-tectum, tegmentum, and cerebellum (Fig. 5F–H). As development proceeds, cerebral expression remains prominent in cerebellum and hindbrain (34–48 hpf) (Fig. 5I–K) while it was absent in somite structures and spinal cord (data not shown).

### 3.3 Characterization of uncx4.1 expression during somite differentiation

**Fig. 6:**
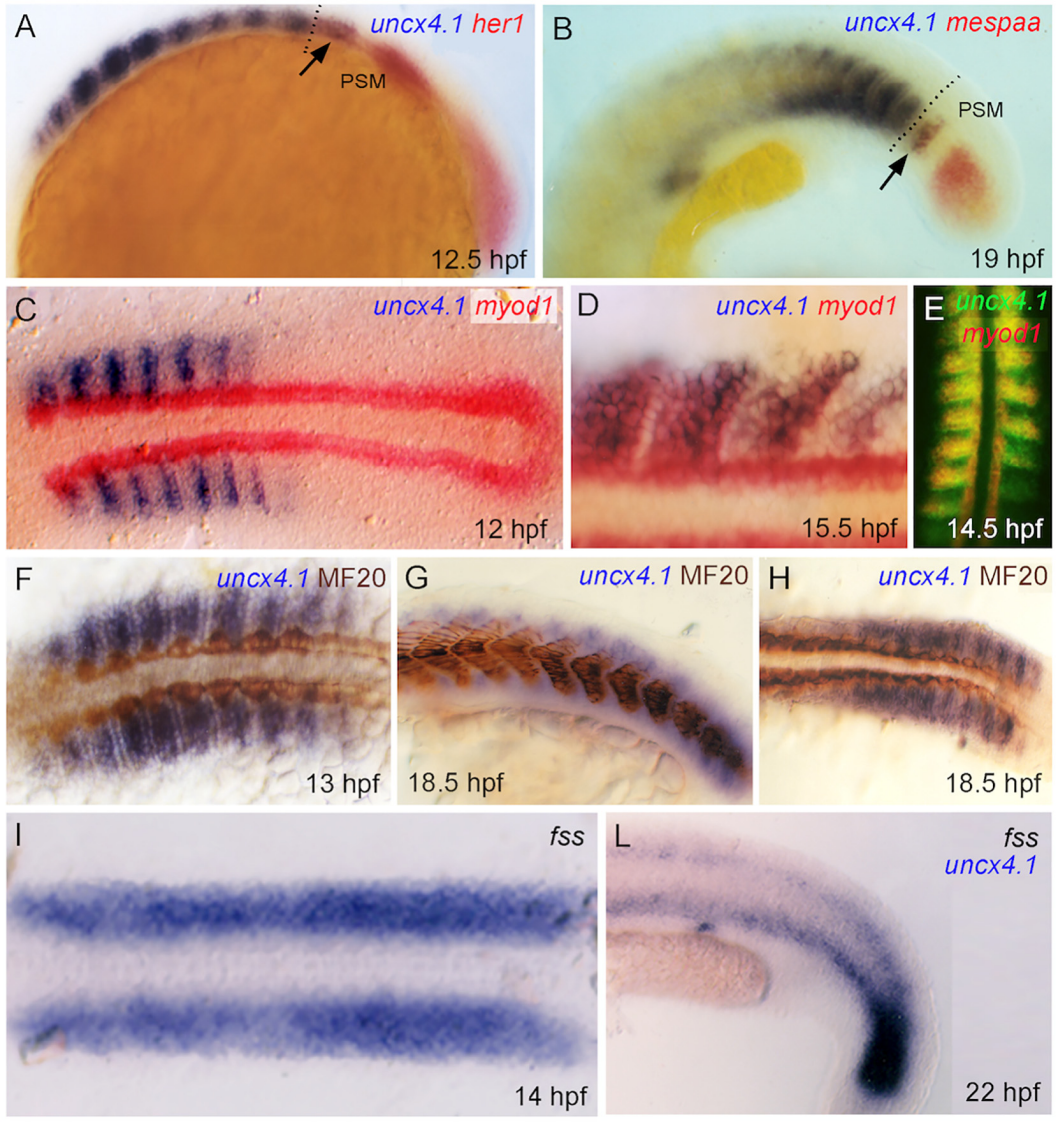
Expression of *uncx4.1* during somite patterning and formation. Whole-mount *in situ* hybridization of *uncx4.1* at (A) 12.5 hours post fertilization (hpf), (B) 19 hpf, (C) 12 hpf, (D, E) 15.5 hpf, (F) 13 hpf, (G, H) 18.5 hpf, (I) 14 hpf, and (L) 22 hpf. (A, B, F, G, L) Lateral view, and (C–F, H, I) dorsal view. (A–D, F–L) Anterior to left, and (E) to top. (A, B) Expression in presomitic mesoderm as shown by double *in situ* labeling with *her1* and *mespaa* riboprobes. PSM = presomitic mesoderm. Dotted lines show boundary between presomitic mesoderm (PSM) and last formed somite. (C–E) Expression is absent in adaxial cells and colocalizes with *myoD1* expression in muscle progenitor cells as shown by double *in situ* labeling with *myoD1* riboprobe at (C) 12 hpf, and (D, E) 15.5 hpf. (F–H) Expression during muscle fibre differentiation as shown by double *in situ* labeling with the MF20 antibody at (F) 13 hpf, and (G, H) 18.5 hpf. (I, L) Expression throughout somitic mesoderm in *fused somite* mutant embryos at (I) 14 hpf, and (L) 22 hpf.

To further explore *uncx4.1* expression during somitogenesis, we carried out double WISH with *her1* and *mespaa*, which revealed that the earliest sign of *uncx4.1* expression occurs in the anterior margin of the PSM and in the anlage of newly forming somite (Fig. 6A, B) (Holley et al., 2000, Holley et al., 2002; Sawada et al., 2000). In paraxial mesoderm, the adaxial cells are the first muscle precursors to express the transcription factor encoding gene *myod1*. These cells migrate laterally to give rise to slow muscle fibers (Devoto et al., 1996). Double hybridization shows that *uncx4.1* mRNA is absent from adaxial cells and is co-expressed with *myod1* in non-adaxial muscle precursor cells at early somitogenesis stages (12 hpf, 6 s) (Fig. 6C–E). Co-labeling of *uncx4.1* mRNA and a pan-myosin antigen (MF20, myosin heavy chain) illustrated how the expansion of myosin signal in maturing muscle cells occurs at the expenses of *uncx4.1* expression in muscle progenitor cells (Fig. 6F–H).

### 3.4 Uncx4.1 expression and somite patterning

To gain understanding as to whether zebrafish *uncx4.1* expression is dependent upon a mechanism of somite antero-posteriorization, we investigated *uncx4.1* expression in segmentless *fused somite* (*fss*^*te314a*^) mutant embryos (*tbx24*), which fail to develop anterior identities within somitic units (Durbin et al., 2000; Windner et al., 2012). As expected, the posteriorization of *fss* somites causes the loss of segmental expression of *uncx4.1*, with uniform transcript distribution throughout the somitic mesoderm (Fig. 6I, L).

As a preliminary assessment of potential roles for *Uncx* genes in somitogenesis in zebrafish, we used morpholino (MO) mediated knock-down of the zebrafish *uncx* genes. Unfortunately, injected *uncx4.1* and *uncx* MOs led to various abnormalities including severe defects in body morphology with defective somitogenesis. Consequently we analyzed muscle differentiation only in those MO-microinjected larvae that were not disturbed in their overall morphology (about 50% *uncx4.1* morphants, 34% *uncx* morphants). Analysis of fast and slow muscle fibers in such 34 hpf embryos injected with transcriptional start site MOs and subsequently stained for F59 (fast and slow MyHCs) and S58 (slow MyHC2) immunostaining shows normally differentiated myoblast cell types in chevron-shaped somites (Suppl. Fig. 6). These results do not allow us to make strong conclusions on potential roles for the *uncx* genes in the somites.

### 3.5 Uncx expression and signaling pathways during somitogenesis

Previous evidence suggests that signaling gradients from the neural tube and notochord-floor plate control the dynamic expression of the *Uncx* gene during somitogenesis (Schrägle et al., 2004). We thus investigated the potential involvement of the Hedgehog, FGF, Notch/Delta and Nodal pathways in directing the spatial expression of zebrafish *Uncx* genes.

#### 3.5.1 Hedgehog

Sonic Hedgehog (Shh) is a signaling molecule secreted by the notochord and floor plate which transduces *via* two transmembrane proteins, Patched 1 (Ptc1) and Smoothened (Smu), and regulates the activity of cubitus interruptus-related (Gli) transcription factors (Borycki et al., 2000). In turn, Gli proteins may act as activators or repressors of Hh signaling (Karlstrom et al., 2003; Tyurina et al., 2005). In zebrafish, the *sonic hedgehog*-*a* (*shha*) gene is expressed in the notochord and floorplate of the neural tube (Krauss et al., 1993).

**Fig. 7:**
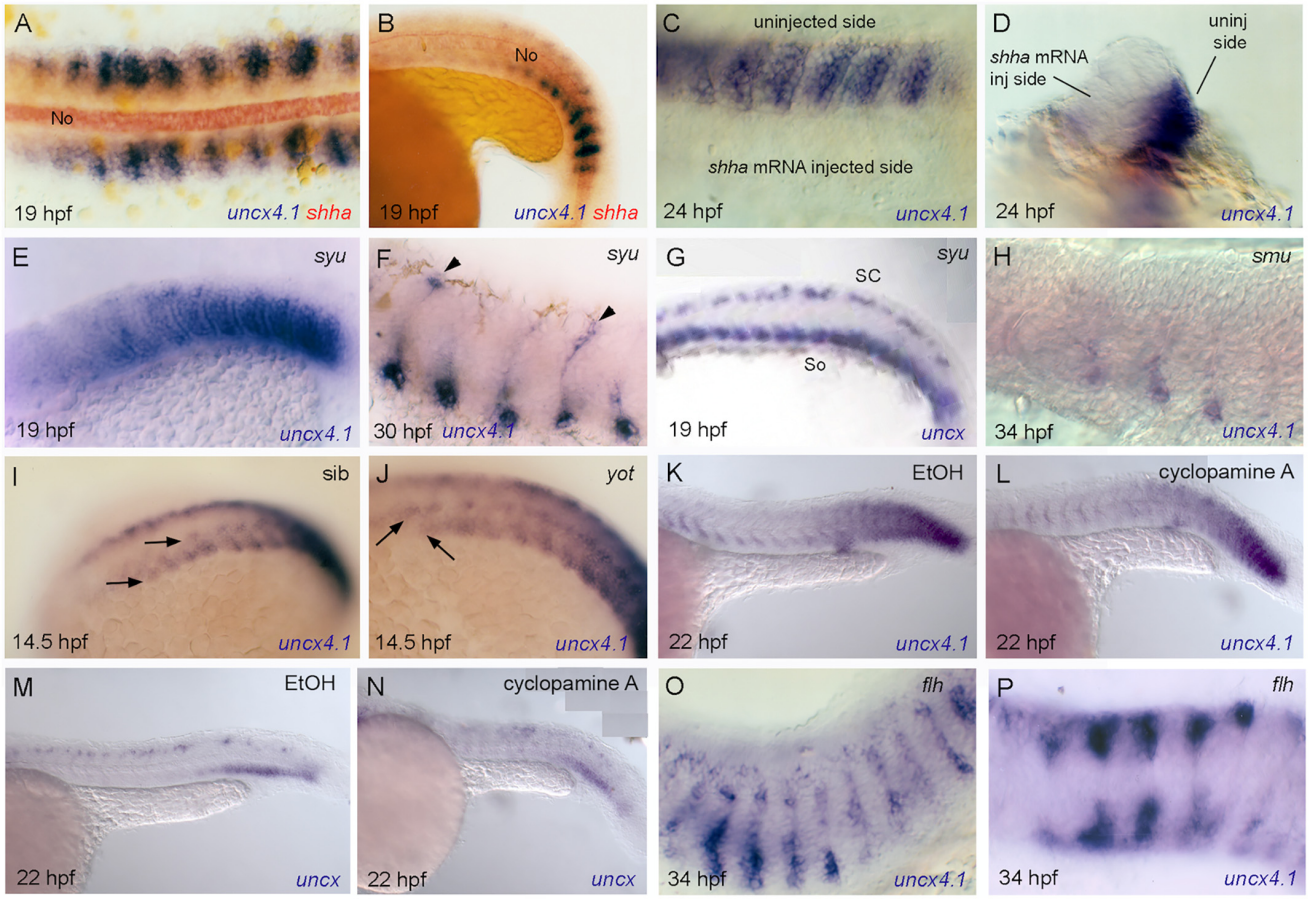
Regulation of *uncx* gene expression by Hedgehog (Hh) signaling pathway. Whole-mount *in situ* hybridization of (A–F, H–L, O, P) *uncx4.1* and (G, M, N) *uncx* at (A, B, E, G) 19 hours post fertilization (hpf), (C, D) 24 hpf, (F) 30 hpf, (H, O, P) 34 hpf, (I, J) 14.5 hpf, and (K–N) 22 hpf. (A, C, P) Dorsal view, (B, E–O) lateral view, and (D) frontal view. (A–C, E–P) Anterior to left, and (D) toward viewer. (A, B) Spatial relationship between *uncx4.1* expression and Hh signal-releasing notochord as shown by double *in situ* labeling with *shha* riboprobe at 19 hpf in (A) dorsal and (B) lateral view. No = notochord. (C, D) Loss of *uncx4.1* expression in *shha* mRNA-injected side at 24 hpf in (C) lateral and (D) frontal view. (E–G) Expression of (E, F) *uncx4.1* and (G) *uncx* in *sonic*-*you* (*syu*) mutant embryos at (E, G) 19 hpf and (F) 30 hpf (controls in Fig. 3E, 5B, and 3H, respectively). (F) Arrowheads indicate *uncx4.1*-expressing cells. SC = spinal cord, So = somites. (H) Expression of *uncx4.1* in *slow*-*muscle*-*omitted* (*smu*) mutant embryos at 34 hpf. (I, J) Expression of *uncx4.1* in (I) sibling (sib) and (J) *you*-*too* (*yot*) mutant embryos at 14.5 hpf. Arrowheads indicate anterior margin of expression in somites. (K–N) Expression of (K, L) *uncx4.1* and (M, N) *uncx* in embryos treated with (K, M) EtOH and (L, N) cyclopamine A at 22 hpf in lateral view. (O, P) Expression of *uncx4.1* in *floating head* (*flh*) mutant embryos at 34 hpf (controls in Fig. 3H and I, respectively).

Our data indicate that *uncx4.1* expression ceases in myogenic progenitor cells localized immediately next to *shha*-expressing tissues (Fig. 7A, B). Furthermore, microinjection of full-length *shha* mRNA abolishes *uncx4.1* expression in zebrafish embryos (Fig. 7C, D), suggesting that Shh may act negatively on *uncx4.1* expression. However, the analysis of several zebrafish mutant embryos in the *Hh* signaling pathway provides a contrasting view of the regulatory role played by Hh signals on *uncx4.1* and *uncx* expression during somitogenesis. The mutant lines studied include *sonic*-*you* (*syu*^*tbx392*^; *sonic hedgehog a*, *shha*) (Brand et al., 1996), *slow*-*muscle*-*omitted* (*smu*^*b577*^; *smoothened*) (Varga et al., 2001), *you*-*too* (*yot*^*ty119*^; *gli2*) (Karlstrom et al., 1999), and *floating head* (*flh*^*n1*^; *noto*) (Melby et al., 1996), the latter mutant lacking *shha*-expressing tissues, *i.e.* notochord and most of the floor plate. In *syu* embryos, *uncx4.1* is still expressed in the myoseptum and dorsal somite cells at 19 and 30–34 hpf, respectively (compare Figs. 3E and 7E, and 3H and 7F). The effect of Shh loss in *syu* embryos seems to be more pronounced in early *uncx* expression, as documented by diffuse mRNA labeling in the ventral portion of the somites at 19 hpf (compare Fig. 5B and F with Fig. 7G). In *smu* embryos, the VLP domain of *uncx4.1* expression is nearly normal (compare Figs. 3H and 7H). The early phase of expression of *uncx4.1* is not significantly altered in *yot* mutant embryos lacking gli2, a dominant repressor of Hh signaling, except for delayed down-regulation in the dorsal domain (Fig. 7I, J). It has already been demonstrated that the level of *ptc* transcripts, a target of Hh signaling, drops by more than half when using 50 μM cyclopamine, a concentration sufficient to impair slow muscle cell differentiation (Wolff et al., 2003). In our hands, inhibition of Hh signaling with cyclopamine does not alter the spatial and temporal transcriptional dynamics of *uncx4.1* and *uncx* when compared with control EtOH-treated embryos (Fig. 7K–N). Finally, *uncx4.1* expression in *flh* mutant embryos is expanded dorsally and medially along the somite posterior boundary with reference to sibling controls (Fig. 7O, P). Since the *flh* mutant lacks the entire notochord and most of the floor plate, this expansion may be the manifestation of a synergic action of different *hh* genes (*i.e.*, *shha*, *shhb*, *ihhb*) (Halpern et al., 1995).

#### 3.5.2 FGF

In the zebrafish, Fgf signaling promotes posterior mesoderm development and border positioning (Sawada et al., 2001). *Acerebellar* (*ace*, *fgf8a*) mutant embryos exhibit only mild somite defects (Reifers et al., 1998; Draper et al., 2003). Groves et al. (2005) have demonstrated that Fgf8a mediates the promotion of a lateral fast muscle fibre population in zebrafish somite. Fgf8a drives *myod1* expression in the lateral posterior stripe of immature caudal somites and is required for the lateral terminal differentiation of fast fibers in maturing rostral somites (Groves et al., 2005).

**Fig. 8:**
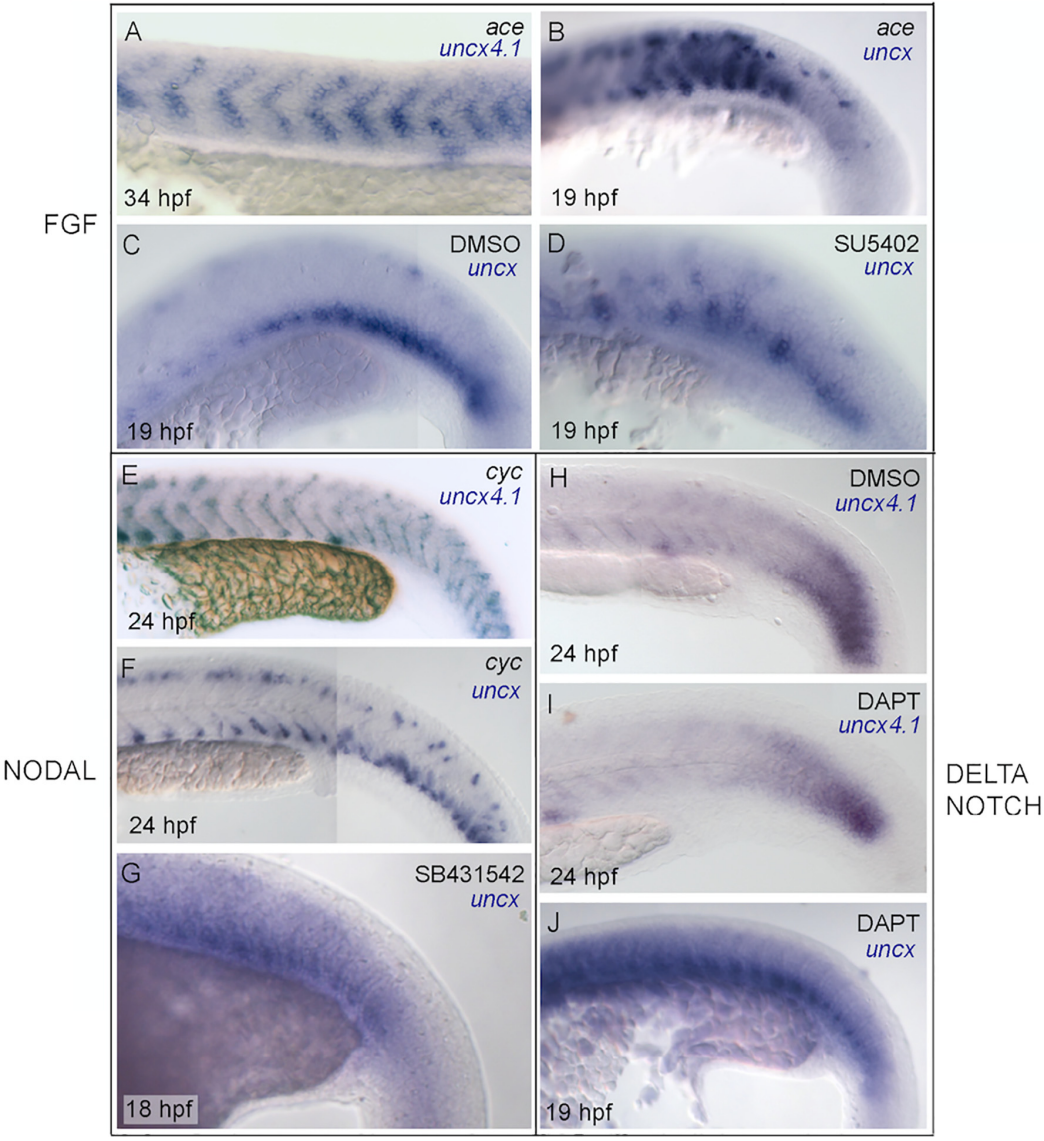
Regulation of *uncx* gene expression in relation to Fgf, Nodal and Notch/Delta signaling pathways. Whole-mount *in situ* hybridization of (A, E, H, I) *uncx4.1* and (B–D, E, G, J) *uncx* at (A) 34 hpf, (B–D) 19 h post fertilization (hpf), (E, F, H) 24 hpf, (G) 18 hpf, and (J) 19 hpf. Lateral view, anterior to left. Expression of (A) *uncx4.1* and (B) *uncx* in *acerebellar* (*ace*) mutant embryo at (A) 34 hpf and (B) 19 hpf. (C, D) Expression of *uncx* in embryos treated with (C) DMSO and (D) SU5402 at 19 hpf. (E, F) Expression of (E) *uncx4.1* and (F) *uncx* in *cyclops* (*cyc*) mutant embryo at 24 hpf (controls in Fig. 3F and 5H, respectively). (G) Expression of *uncx* in embryo treated with SB431542 at 18 hpf (control in Fig. 5B). (H–J) Expression of (H, I) *uncx4.1* and (J) *uncx* in embryos treated with (H) DMSO and (I, J) DAPT at (H, I) 24 hpf and (J) 19 hpf. (J) Control in (C).

*fgf8a* mutant embryos (*acerebellar*, *ace*^*ti282a*^) (Reifers et al., 1998) were thus used to explore the possible contribution of FGF signaling in the regulation of *uncx* genes during somite development. Both *uncx4.1* and *uncx* fail to confine ventrally in *ace* homozygote embryos, suggesting that Fgf8a negatively regulates the expression of *uncx4.1* (compare Figs. 3H and 8A for *uncx4.1*; Figs. 5B and 8B for *uncx*). Accordingly, the expression of *uncx* in embryos exposed to the Fgf inhibitor SU5402 is disrupted and partially dorsally expanded compared with control embryos treated with DMSO vehicle (compare Fig. 8C with Fig. 8D).

#### 3.5.3 Nodal

A conserved role for Nodal factors, belonging to the TGFβ family, has been proposed in the formation of the mesoderm (Harland and Gerhart, 1997; Hagos and Dougan, 2007). The dynamic expression of *uncx4.1* and *uncx* was largely unchanged during somite formation in the *cyclops* mutant (*cyc*^*b16*^; *nodal*-*related protein*, *ndr2*) (Rebagliati et al., 1998) (compare Fig. 3F with Fig. 8E, and Fig. 5H with Fig. 8F). Similarly, *uncx* expression was normal in embryos treated with SB431542, an inhibitor of Nodal signaling (compare Fig. 8C with Fig. 8G).

#### 3.5.4 Notch/Delta

In chi9ck, it has been proposed that Notch/Delta signaling induces *Uncx* transcription in the cranial PSM (Schrägle et al., 2004). We aimed to verify if the regulatory interaction between Notch/Delta driven oscillator activity and *Uncx* gene expression suggested in birds is conserved in teleosts. Previous data indicate that pharmacological blockade of the Notch/Delta pathway in zebrafish, by using the gamma-secretase inhibitor DAPT, induces somite defects only after long developmental delays, suggesting that Notch/Delta signaling is essential for synchronizing oscillations of neighboring cells in the posterior PSM but not for somite border formation (Mara et al., 2007; Özbudak and Lewis, 2008; Sewell et al., 2009). We found that the expression of *uncx4.1* and *uncx* in zebrafish embryos treated with DAPT is similar to that observed in control DMSO-treated embryos (compare Fig. 8H with Fig. 8I, and compare Fig. 8C with Fig. 8J, respectively).

### 3.6 Uncx4.1 and axogenesis

*C. elegans Unc*-*4* is well known for its role in axonal connections, acting as a determinant of synaptic choice for motor neurons (Schneider et al., 2012). In this study, we observed ancient syntenic association between *Uncx* and two genes involved in axogenesis (a *Mical* gene, *micall2*) and synaptic choice (*Elfn1*). Looking for correlations between *Uncx* gene expression and axon guidance, we first performed double labeling with the primary motor axon marker *znp1*, finding that the outgrowth of the caudal primary (CaP) motor axons coincides with the progressive down-regulation of *uncx4.1* expression during somite development (Suppl. Fig. 7A, B, G). Then, we observed that the *netrin*-*1b* (*ntn1b*) gene, a member of a secreted protein family mediating axon guidance, is expressed in VLP cells at 34 hpf (Suppl. Fig. 7C, D–F, and Fig. 3I with Suppl. Fig. 7F). Finally, *uncx4.1* over-expressing embryos display marked up-regulation of *ntn1b* with stunted and prematurely branching CaP axons, a phenotype possibly caused by surrounding the motor neuron growth cone with cells ectopically expressing the chemoattractant *ntn1b* (Suppl. Fig. 7H–K).

## 4 Discussion

### 4.1 Origin and evolution of the Uncx genes

Since Metazoan Uncx proteins are poorly characterized from an evolutionary perspective (Woolfe and Elgar, 2007; Sánchez and Sánchez, 2013), we provided a comprehensive phylogenetic reconstruction showing the orthology of all analyzed genes, which we refer to as *Uncx* (Fig. 1). The partial protein-coding sequence found in the cnidarian *N. vectensis* genome (Suppl. File 2) as well as the absence of *Uncx* in sponges (Porifera), suggest that this homeobox gene was already present in the ancestor of bilaterians, though with instances of gene duplication and/or gene loss (Fig. 2). A common origin for *Uncx* genes is confirmed by the conservation of intron/exon structure in Bilateria (Suppl. File 3). We highlighted duplications in unrelated invertebrate taxa (*i.e.*, *D. melanogaster*, *C. teleta*, *S. kowalevskii*, *C. robusta*) and the absence of *Uncx* in early branching metazoans (*e.g.*, Placozoa and Ctenophora), in the agnathan lamprey and in many reptiles, which may underly functional gene diversification, with loss or (re)gain of (ancestral) gene functions (Albalat and Cañestro, 2016). As proposed for *T. rubripes* (Woolfe and Elgar, 2007), we report that teleosts have two *Uncx* duplicates, currently known as *Uncx4.1* and *Uncx*. The retention of both co-orthologs reflects the over-representation of duplicated transcription factors in fish genomes (Roest Crollius and Weissenbach, 2005), depending on key roles in development and cellular differentiation. Interestingly, two rounds of whole-genome duplications (WGDs) at the stem of vertebrates (Ohno, 1993; Abi-Rached et al., 2002; Dehal and Boore, 2005) imply the presence of other three *Uncx* members in gnathostome ancestor, which have been secondarily lost during evolution.

We sought to provide insights into *Uncx* molecular evolution by analysing its genomic *locus* from cnidarians to human. It has been reported the presence of almost 800 conserved ancestral microsyntenic pair (CAMP) combinations for several homeobox genes as *Uncx* from cnidarians as *Nematostella* to cephalochordates as *Branchiostoma* (Irimia et al., 2012). We found that the *Uncx* gene forms distinct microsyntenic clusters. An invertebrate CAMP with the transcription factor encoding *Alx*/*Cart*-1 gene is seen in annelids and hemichordates (Suppl. Fig. 3), while in surveyed Olfactores, *Uncx* orthologs are coupled with *Elfn1* (Suppl. Fig. 2). A cluster formed by *Uncx*, *Elfn1*, and *Micall2* genes exists in gnathostomes, which is also duplicated in teleosts (Fig. 2). The conserved chromosomal vicinity of *Uncx* and *Micall2* genes evokes a “bystander interference effect” exerted by one of the two genes, which has been proposed for genes implicated in key developmental mechanisms (Cajiao et al., 2004).

VISTA comparison of *Uncx* loci belonging to mollusks, brachiopods, ascidians, and vertebrates indicates conservation of sequence, consistent with past studies on *Uncx* highlighting the presence of CNEs (conserved non-coding elements) in *Takifugu rubripes* and *Homo sapiens* (Woolfe and Elgar, 2007) (Suppl. Fig. 5). Teleost *Uncx* paralogs lack some of the conserved elements common among coelacanth, spotted gar and human, whose lineages diverged from teleosts before the TSGD (Suppl. Fig. 5). In addition, they exhibit differences in peak patterns as if had undergone an asymmetrical rate of evolution.

The expression patterns of the two *Uncx* paralogous genes show unique (*uncx4.1*: pharyngeal arches and kidney; *uncx*: spinal cord) and partially overlapping domains (CNS and somites). These findings are possibly associated with genome duplication producing divergent regulatory modality, with events of subfunctionalization and/or neofunctionalization. However, the potential for cross-hybridisation needs to be considered when working with paralogous genes. In our work, divergent hybridization patterns with high signal and low background riboprobes were obtained, indicative of high levels of specificity and minimal cross-hybridization between duplicated genes.

The analysis of vertebrate genome environment demonstrated that *Uncx4.1* and *Uncx* genes descend from the same paralogon (Fig. 2); therefore, they derive from the teleost-specific genome duplication (TSGD), which occurred 300–350 million of years ago (Taylor et al., 2001; Taylor et al., 2003; Jaillon et al., 2004; Kuraku and Meyer, 2009). In light of the above, we propose to change the name of teleost *Uncx* paralogs genes into *uncxa* (*Uncx4.1*; NM_001020780.2) and *uncxb* (*uncx*; XM_005164204.4).

### 4.2 Regulation of the zebrafish uncx genes

In this study, *uncx4.1* gene co-expression in the anterior presomitic mesoderm (PSM) with Notch-pathway gene *her1*, the output of the molecular clock, and with the Mesp1-related factor encoding *mespaa* (Fig. 6A, B), suggests that zebrafish *uncx* genes are controlled by players in somite anterior-posterior specification. This observation also indicates that, similarly to what is observed in mouse, zebrafish *Uncx* genes could be required for maintaining antero-posterior polarity within the somite (Farin et al., 2008; Lee et al., 2009). However, it is worth to note that the murine *Uncx* gene is expressed in the posterior half of the newly formed somites but, unlike the fish and chick orthologs, it is not active in the PSM (Barrantes et al., 1999; Schrägle et al., 2004). Furthermore, we show that, as in the zebrafish *mesp* quadruple mutant, *uncx4.1* expression is extended to the entire somite of *fused somite*/*tbx6* mutant embryos (Fig. 6I, L) (Yabe et al., 2016). Considering that mouse *Tbx6* is involved in somite boundary positioning together with *Mesp*, and that *Mesp* provides positional information within the somite, a similar mechanism to induce zebrafish *uncx* gene expression in the caudal somite half may occur during the establishment of somite polarity and boundary formation.

In zebrafish, the somite develops into a large myotome, with a smaller group of ventral cells specified as sclerotome (Stickney et al., 2000). Genes encoding myogenic regulatory factors such as *myod1* and *myf5* are expressed early in the most medial presomitic mesoderm adjacent to the notochord (Devoto et al., 1996; Weinberg et al., 1996). Both *myod* and *myf5* control the commitment to the myogenic lineage and are required for the initiation of the *myogenin* gene expression (Pownall et al., 2002). During early somitogenesis in zebrafish, *uncx4.1* activation coincides with that of *myod1* in muscle progenitor cells (Fig. 6C, D), indicating that zebrafish *Uncx* paralogues may function in somites at the onset of muscle differentiation. The absence of *uncx4.1* and *uncx* gene expression in adaxial cell precursors adjacent to the notochord (Fig. 6C–E) suggests that *uncx* genes are not required for the specification and differentiation of slow muscle cells.

During somite formation, the distribution of *uncx4.1* and *uncx* transcripts becomes progressively confined to a small population of undifferentiated myoblasts at the ventral lateral posterior (VLP) margin (Fig. 3, Fig. 6). VLP cells expressing *Uncx* genes are likely connected to an extended ventral monolayer termed growth zone, which is known to contribute to hyperplastic growth of each myotome in marine teleosts (Barresi et al., 2000, Barresi et al., 2001). In this view, *uncx4.1* could inhibit muscle formation *via* induction of myoblast proliferation at the expenses of muscle differentiation and/or as an antagonist of late differentiation (Fig. 6F–H).

We attempted to place *uncx* genes in the context of signal transduction mechanisms (*i.e.*, Hh, FGF, Notch/Delta, Nodal) already known to play key roles in somite patterning and differentiation in zebrafish. Hh signal transduction is an intricate molecular pathway that acts in a dosage-dependent manner to specify cell fate in the zebrafish myotome (Wolff et al., 2003). The expression of *uncx4.1* is lost in the Hh pathway component *ptc1*; *ptc2* mutants (Koudijs et al., 2008). However, our data do not clarify whether or not Hedgehog signaling is required to drive expression of *uncx4.1* and *uncx*; and, if so, to which extent. Also, the analysis of the regulatory interactions between *Uncx* genes and the Notch/Delta and Nodal pathways do not provide conclusive results with only changes to *uncx* expression. Accordingly, previous evidence in Notch1 mutant mouse shows that *Uncx* expression is slightly wider than in sibling embryos but essentially unaltered (Barrantes et al., 1999). Finally, somite expression of zebrafish *Uncx* genes in Fgf8a mutant embryos and in embryos treated with the Fgf inhibitor SU5402 is disrupted and dorsally extended, consistent with a negative role played by Fgf signaling in the expression of *Uncx* genes in zebrafish somitogenesis. The relationship between *Uncx* expression and fast muscle fibers warrants more careful examination in zebrafish *fgf8a* mutants. When all our evidence is considered, it suggests a hypothesis whereby *Uncx* gene expression is specifically regulated by Fgf signaling, while Hh, Notch/Delta and Nodal signals may have more subtle roles in controlling the dynamic pattern of *Uncx* expression during somitogenesis.

### 4.3 A dual role in somitogenesis and axon guidance?

**Fig. 9:**
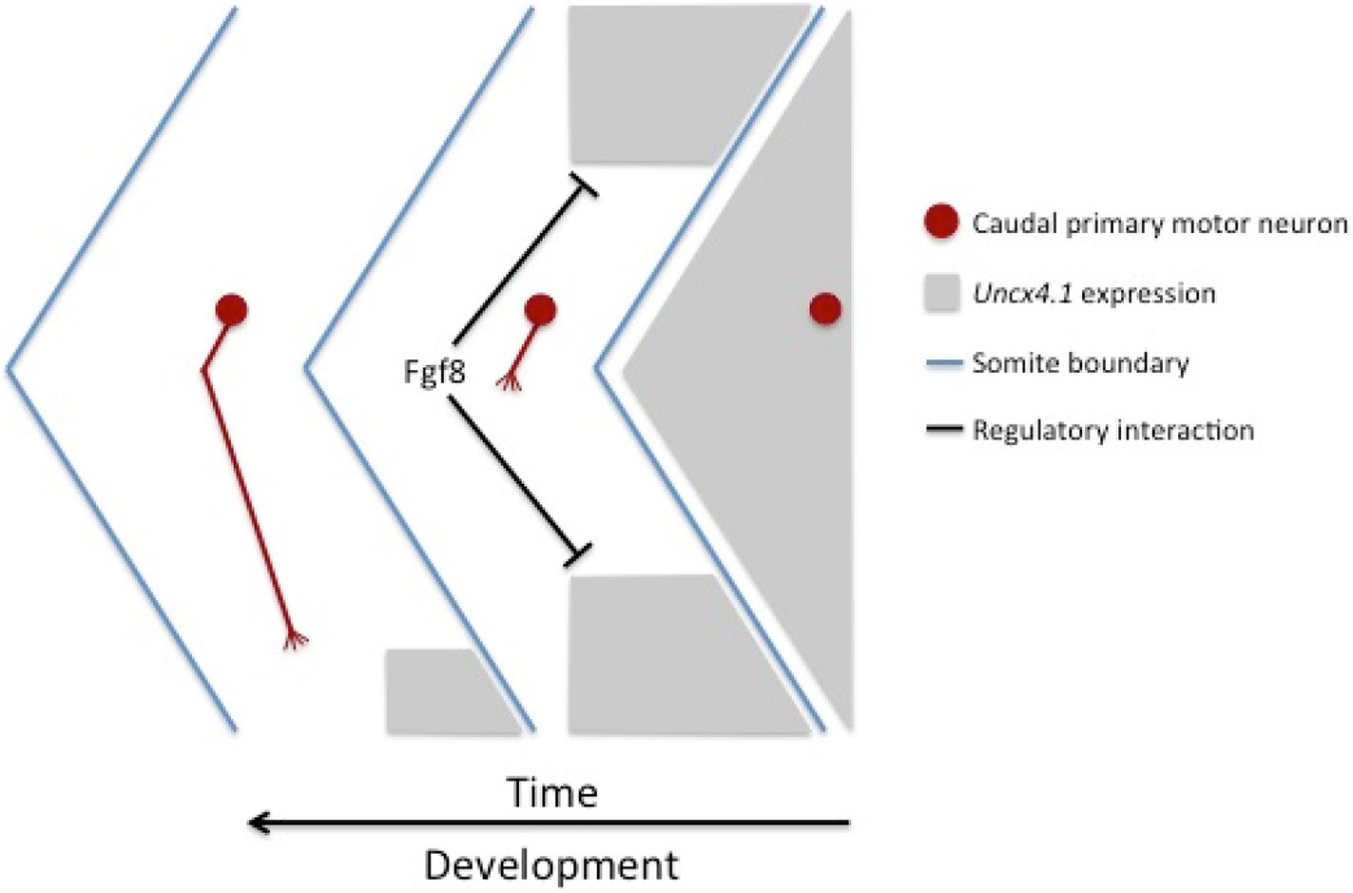
Scheme of zebrafish *uncx4.1* gene expression and regulation during somitogenesis.

*In silico* analysis of available genome databases revealed the physical co-localization of *Uncx* with genes implicated in synaptic functioning and plasticity, *i.e. Micall2* (gnathostome-specific gene duplet). Also, a correlation was observed between the expression patterns of *uncx4.1* and *ntn1b*, a member of a secreted protein family mediating axon guidance, and the trajectory of caudal primary (CaP) motor neuron axons. While Netrin is an attractant cue in *Drosophila* axon guidance (Hiramoto and Hiromi, 2006; Brankatschk and Dickson, 2006), the role of its zebrafish ortholog is not completely resolved, even if a diffuse *ntn1b* expression within the somite is thought to promote ventral elongation of the CaP motor axon (Hale et al., 2011). The ventral restriction of *uncx4.1* and *ntn1b* expression might involve a mechanism comprising the release of positional signals that contribute to the restriction of the CaP axon pathways. This may occur either by attracting CaP axons by diffusion of chemoattractants across intersomitic boundary epithelia, like in *Drosophila*, or repelling them within each somite through long-range cues (Mitchell et al., 1996). The CaP axon phenotype induced by *uncx4.1* mRNA injection is similar to the effects of ectopically expressed netrins in other systems (*Drosophila*) (Mitchell et al., 1996). We speculate that the zebrafish Uncx4.1 activity in a particular subset of myotomal cells might serve a dual function by interacting with cell-cycle genes in controlling cell divisions during myoblast differentiation, and by activating or maintaining *ntn1b* expression for proper axonal elongation (Fig. 9).

## Conflicts of interest

The authors declare that they have no conflict of interest.

## Appendix A Supplementary data

Supplemental File 1 FASTA file comprising protein sequences employed for Uncx phylogeny. Supplemental File 2 FASTA file including protein sequences excluded from Uncx phylogeny due to their molecular divergence. Supplemental File 3 Alignment of Uncx proteins showing the presence of a conserved intron/exon structure. Color-coded text highlights conserved splicing sites. Supplemental Fig. 1 Corresponding position of riboprobes. Red line: riboprobe. Red rectangle: exon. Orange rectangle: homeobox. White rectangle: untranslated sequence. Numbers indicate nucleotide sequence length. Supplemental Fig. 2 Synteny conservation of *Uncx* genes in Olfactores. Horizontal bars represent orthologous genomic regions of man (*H. sapiens*, Hsa7), ascidian (*C. robusta*, Cro11) and zebrafish (*D. rerio*, Dre3 and Dre1). Orthologous genes are shown with same colors. Lines highlight a conserved microsynteny among *Uncx* and *Elfn1* genes. Supplemental Fig. 3 Syntenic conservation of *Uncx* genes in *Metazoa*. Horizontal lines represent orthologous genomic regions of annelid polychaete (*C. teleta*, Sc658: 20,810–22,694), insect (*D. melanogaster*, Chr X), hemichordate (*S. kowalevskii*, NW_003125889.1), amphioxus (*B. floridae*, NW_003101431.1), and human (*H. sapiens*, Hsa7). Orthologous genes are shown with same colors. Supplemental Fig. 4 VISTA plot of the intergenic space between *Uncx* and *Micall2* genes in *H. sapiens* and *D. rerio*. Supplemental Fig. 5 VISTA analysis of *Uncx* gene *loci* in Metazoans. Exons are highlighted in violet and UTR regions in light blue, while conserved non-coding elements (CNE) are shown in pink. Horizontal lines showing peaks of similarity in pairwise sequence alignments between human *Uncx locus vs.* orthologous genomic regions of mollusk (*C. gigas*, Cg), brachiopod (*L. anatina*, La), ascidian (*C. robusta*, Cr), coelacanth (*L. chalumnae*, Lc), and actinopterygians (*L. oculatus*, Lo; *D. rerio*, Dr; *T. nigroviridis*, Tn). Orange and green boxes highlight lack of conserved regions upstream and in the second intron of teleosts, respectively. Blue box denotes sequence divergence in the upstream region between teleost paralogous genes. Supplemental Fig. 6 Knock-down of zebrafish *uncx* genes. Immunohistochemistry of knock-down and control embryos at 34 h post fertilization (hpf) with (A, B) MF20, (C, D) F59 (24 hpf), and (G, H) S58 antibodies. CtrMO = standard control morpholino. Anterior to left, dorsal to top. Supplemental Fig. 7 Expression of *uncx4.1* in relation to axon guidance. (A–F, H, I) Whole-mount *in situ* hybridization of (A, B) *uncx4.1*, and (C–F) *ntn1b* at (A) 19 hours post fertilization (hpf), (B) 30 hpf, (C) 13.5 hpf, (D) 22 hpf, (E–G, J, K) 34 hpf, and (H, I) 24 hpf. (G, J, K) Whole-mount immunohistochemistry to label motor neuron axons with the znp1 antibody. (A, B, D, E, J, K) lateral view, (C, H) dorsal view, and (F, G, I) frontal view. (A–E, H, J, K) Anterior to left, or (F, G, I) toward viewer. (A, B) Expression with respect to (A) axonal outgrowth (black arrowheads) of primary caudal (CaP) motor neurons and (B) elongation (asterisks) at (A) 19 hpf and (B) 30 hpf. Dotted lines indicate boundaries between somites. SC = spinal cord. (C–F) Expression of *ntn1b* (C) 13.5 hpf, (D) 22 hpf, and (E, F) 34 hpf. (D) White arrow indicates lack of expression in ventro-latero-posterior cells (VLP) of younger somites; black arrow indicates expression in VLP cells of older somites. (E) Dotted lines indicate boundaries between somites. (F) Arrowhead indicates expression in VLP cells of each somite. PSM = presomitic mesoderm, SC = spinal cord, So = somite, No = notochord. (G) Primary motor neuron axon pathfinding in transversal section at 34 hpf. (H, I) *ntn1b* expression in *uncx4.1* mRNA-injected embryos at 24 hpf (control in Suppl. Fig. 5E). (J, K) Abnormally branching CaP axons (white arrowheads) in (K) *uncx4.1* mRNA-injected embryos compared to (J) un-injected ones. CaP = caudal primary motor neuron axons. Image 1

## References

[bb0005] Abi-Rached L., Gilles A., Shiina T., Pontarotti P., Inoko H. (2002). Evidence of en bloc duplication in vertebrate genomes. Nat. Genet..

[bb0010] Albalat R., Cañestro C. (2016). Evolution by gene loss. Nat. Rev. Genet..

[bb0015] Asbreuk C.H., van Doorninck J.H., Mansouri A., Smidt M.P., Burbach J.P. (2006). Neurohypophysial dysmorphogenesis in mice lacking the homeobox gene Uncx4.1. J. Mol. Endocrinol..

[bb0025] Barrantes I.B., Elia A.J., Wünsch K., De Angelis M.H., Mak T.K., Rossant J., Conlon R.A., Gossler A., de la Pompa J.L. (1999). Interaction between Notch signalling and Lunatic fringe during somite boundary formation in the mouse. Curr. Biol..

[bb0030] Barresi M.J., Stickney H.L., Devoto S.H. (2000). The zebrafish slow-muscle-omitted gene product is required for Hedgehog signal transduction and the development of slow muscle identity. Development.

[bb0035] Barresi M.J.F., D'Angelo J.A., Hernández L.P., Devoto S.H. (2001). Distinct mechanisms regulate slow-muscle development. Curr. Biol..

[bb0045] Borycki A.G., Brown A.M.C., Emerson C.P. (2000). Shh and Wnt signaling pathways converge to control Gli gene activation in avian somites. Development.

[bb0050] Brand M., Heisenberg C.P., Warga R.M., Pelegri F., Karlstrom R.O., Beuchle D., Nüsslein-Volhard C. (1996). Mutations affecting development of the midline and general body shape during zebrafish embryogenesis. Development.

[bb0055] Brankatschk M., Dickson B.J. (2006). Netrins guide *Drosophila* commissural axons at short range. Nat. Neurosci..

[bb0060] Bussen M., Petry M., Schuster-Gossler K., Leitges M., Gossler A., Kispert A. (2004). The T-box transcription factor Tbx18 maintains the separation of anterior and posterior somite compartments. Genes Dev..

[bb0065] Cajiao I., Zhang A., Yoo E.J., Cooke N.E., Liebhaber S.A. (2004). Bystander gene activation by a locus control region. EMBO J..

[bb0070] Cao Y., Sarria I., Fehlhaber K.E., Kamasawa N., Orlandi C., James K.N., Martemyanov K.A. (2015). Mechanism for selective synaptic wiring of rod photoreceptors into the retinal circuitry and its role in vision. Neuron.

[bb0075] Catchen J.M., Conery J.S., Postlethwait J.H. (2009). Automated identification of conserved synteny after whole genome duplication. Genome Res..

[bb0090] Daniele G., Simonetti G., Fusilli C., Iacobucci I., Lonoce A., Palazzo A., Storlazzi C.T. (2017). Epigenetically induced ectopic expression of UNCX impairs the proliferation and differentiation of myeloid cells. Haematologica.

[bb0095] Dehal P., Boore J.L. (2005). Two rounds of whole genome duplication in the ancestral vertebrate. PLoS Biol..

[bb0105] Devoto S.H., Melançon E., Eisen J.S., Westerfield M. (1996). Identification of separate slow and fast muscle precursor cells in vivo, prior to somite formation. Development.

[bb0115] Draper B.W., Stock D.W., Kimmel C.B. (2003). Zebrafish fgf24 functions with fgf8 to promote posterior mesodermal development. Development.

[bb0125] Durbin L., Sordino P., Barrios A., Gering M., Thisse C., Thisse B., Brennan C., Green A., Wilson S., Holder N. (2000). Anteroposterior patterning is required within segments for somite boundary formation in developing zebrafish. Development.

[bb0130] Farin H.F., Mansouri A., Petry M., Kispert A. (2008). T-box protein Tbx18 interacts with the paired box protein Pax3 in the development of paraxial mesoderm. J. Biol. Chem..

[bb0135] Gertz E.M., Yu Y.K., Agarwala R., Schaffer A.A., Altschul S.F. (2006). Composition-based statistics and translated nucleotide searches: improving the TBLASTN module of BLAST. BMC Biol..

[bb0140] Giot L., Bader J.S., Brouwer C., Chaudhuri A., Kuang B., Li Y., Rothberg J.M. (2003). A protein interaction map of *Drosophila melanogaster*. Science.

[bb0145] Gordon D.F., Wagner J., Atkinson B.L., Chiono M., Berry R., Sikela J., Gutierrez-Hartmann A. (1996). Human Cart-1: structural organization, chromosomal localization, and functional analysis of a cartilage-specific homeodomain cDNA. DNA Cell Biol..

[bb0150] Groves J.A., Hammond C.L., Hughes S.M. (2005). Fgf8 drives myogenic progression of a novel lateral fast muscle fibre population in zebrafish. Development.

[bb0155] Hagos E.G., Dougan S.T. (2007). Time-dependent patterning of the mesoderm and endoderm by nodal signals in zebrafish. BMC Dev. Biol..

[bb0160] Hale L.A., Fowler D.K., Eisen J.S. (2011). Netrin signaling breaks the equivalence between two identified zebrafish motoneurons revealing a new role of intermediate targets. PLoS One.

[bb0165] Halpern M.E., Thisse C., Ho R.K., Thisse B., Riggleman B., Trevarrow B., Kimmel C.B. (1995). Cell-autonomous shift from axial to paraxial mesodermal development in zebrafish floating head mutants. Development.

[bb0170] Harland R., Gerhart J. (1997). Formation and function of Spemann's organizer. Annu. Rev. Cell Dev. Biol..

[bb0175] Hiramoto M., Hiromi Y. (2006). ROBO directs axon crossing of segmental boundaries by suppressing responsiveness to relocalized netrin. Nat. Neurosci..

[bb0180] Holley S.A., Geisler R., Nüsslein-Volhard C. (2000). Control of her1 expression during zebrafish somitogenesis by a Delta-dependent oscillator and an independent wave-front activity. Genes Dev..

[bb0185] Holley S.A., Jülich D., Rauch G., Geisler R., Nüsslein-Volhard C. (2002). her1 and the notch pathway function within the oscillator mechanism that regulates zebrafish somitogenesis. Development.

[bb0190] Huson D.H., Scornavacca C. (2012). Dendroscope 3: an interactive tool for rooted phylogenetic trees and networks. Syst. Biol..

[bb0195] Irimia M., Tena J.J., Alexis M.S., Fernandez-Miñan A., Maeso I., Bogdanović O., de la Calle-Mustienes E., Roy S.W., Gómez-Skarmeta J.L., Fraser H.B. (2012). Extensive conservation of ancient microsynteny across metazoans due to cis-regulatory constraints. Genome Res..

[bb0200] Jaillon O., Aury J.M., Brunet F., Petit J.L., Stange-Thomann N., Mauceli E., Roest Crollius H. (2004). Genome duplication in the teleost fish *Tetraodon nigroviridis* reveals the early vertebrate proto-karyotype. Nature.

[bb0205] Junker J.P., Noël E.S., Guryev V., Peterson K.A., Shah G., Huisken J., van Oudenaarden A. (2014). Genome-wide RNA tomography in the zebrafish embryo. Cell.

[bb0210] Karlstrom R.O., Talbot W.S., Schier A.F. (1999). Comparative synteny cloning of zebrafish you-too: mutations in the Hedgehog target gli2 affect ventral forebrain patterning. Genes Dev..

[bb0215] Karlstrom R.O., Tyurina O.V., Kawakami A., Nishioka N., Talbot W.S., Sasaki H., Schier A.F. (2003). Genetic analysis of zebrafish gli1 and gli2 reveals divergent requirements for gli genes in vertebrate development. Development.

[bb0220] Kimmel C.B., Ballard W.W., Kimmel S.R., Ullmann B., Schilling T.F. (1995). Stages of embryonic development of the zebrafish. Dev. Dyn..

[bb0225] Koudijs M.J., den Broeder M.J., Groot E., van Eeden F.J.M. (2008). Genetic analysis of the two zebrafish patched homologues identifies novel roles for the hedgehog signaling pathway. BMC Dev. Biol..

[bb0230] Kozak M. (1986). Point mutations define a sequence flanking the AUG initiator codon that modulates translation by eukaryotic ribosomes. Cell.

[bb0235] Krauss S., Concordet J.P., Ingham P.W. (1993). A functionally conserved homolog of the *Drosophila* segment polarity gene hh is expressed in tissues with polarizing activity in zebrafish embryos. Cell.

[bb0240] Krieg P.A., Melton D.A. (1984). Functional messenger RNAs are produced by SP6 in vitro transcription of cloned cDNAs. Nucleic Acids Res..

[bb0245] Kuraku S., Meyer A. (2009). The evolution and maintenance of Hox gene clusters in vertebrates and the teleost-specific genome duplication. Int. J. Dev. Biol..

[bb0250] Lee H.C., Tseng W.A., Lo F.Y., Liu T.M., Tsai H.J. (2009). FoxD5 mediates anterior-posterior polarity through upstream modulator Fgf signaling during zebrafish somitogenesis. Dev. Biol..

[bb0255] Leitges M., Neidhardt L., Haenig B., Herrmann B.G., Kispert A. (2000). The paired homeobox gene Uncx4.1 specifies pedicles, transverse processes and proximal ribs of the vertebral column. Development.

[bb0260] Mansouri A., Yokota Y., Wehr R., Copeland N.G., Jenkins N.A., Gruss P. (1997). Paired-related murine homeobox gene expressed in the developing sclerotome, kidney, and nervous system. Dev. Dyn..

[bb0265] Mansouri A., Voss A.K., Thomas T., Yokota Y., Gruss P. (2000). Uncx4.1 is required for the formation of the pedicles and proximal ribs and acts upstream of Pax9. Development.

[bb0270] Mara A., Schroeder J., Chalouni C., Holley S.A. (2007). Priming, initiation and synchronization of the segmentation clock by deltaD and deltaC. Nat. Cell Biol..

[bb0275] Melby A.E., Warga R.M., Kimmel C.B. (1996). Specification of cell fates at the dorsal margin of the zebrafish gastrula. Development.

[bb0280] Miller D.M., Niemeyer C.J. (1995). Expression of the *unc*-*4* homeoprotein in *Caenorhabditis elegans* motor neurons specifies presynaptic input. Development.

[bb0285] Miller D.M., Shen M.M., Sham C.E., Bürglin T.R., Ruvkun G., Dubois M.L., Ghee M., Wilson L. (1992). C. elegans *unc*-*4* gene encodes a homeodomain protein that determines the pattern of synaptic input to specific motor neurons. Nature.

[bb0290] Mitchell K.J., Doyle J.L., Serafini T., Kennedy T.E., Tessier-Lavigne M., Goodman C.S., Dickson B.J. (1996). Genetic analysis of *Netrin* genes in *Drosophila*: netrins guide CNS commissural axons and peripheral motor axons. Neuron.

[bb0295] Morin-Kensicki, Eisen E.M. (1997). Sclerotome development and peripheral nervous system segmentation in embryonic zebrafish. Development.

[bb0300] Nakajima Y., Morimoto M., Takahashi Y., Koseki H., Saga Y. (2006). Identification of Eph4 enhancer required for segmental expression and the regulation by Mesp2. Development.

[bb0305] Neidhardt L.M., Kispert A., Herrmann B.G. (1997). A mouse gene of the paired-related homeobox class expressed in the caudal somite compartment and in the developing vertebral column, kidney and nervous system. Dev. Genes Evol..

[bb0310] Ohno S. (1993). Patterns in genome evolution. Curr. Opin. Genet. Dev..

[bb0315] Özbudak E.M., Lewis J. (2008). Notch signalling synchronizes the zebrafish segmentation clock but is not needed to create somite boundaries. PLoS Genet..

[bb0320] Pflugrad A., Meir J.Y., Barnes T.M., Miller D.M. (1997). The Groucho-like transcription factor UNC-37 functions with the neural specificity gene unc-4 to govern motor neuron identity in *C. elegans*. Development.

[bb0325] Pownall M.E., Gustafsson M.K., Emerson C.P. (2002). Myogenic regulatory factors and the specification of muscle progenitors in vertebrate embryos. Annu. Rev. Cell Dev. Biol..

[bb0330] Rabe T., Griesel G., Blanke S., Kispert A., Leitges M., van der Zwaag B., Burbach J.P.H., Varoqueaux F., Mansouri A. (2012). The transcription factor Uncx4.1 acts in a short window of midbrain dopaminergic neuron differentiation. Neural Dev..

[bb0335] Ratnere I., Dubchak I. (2009). Obtaining comparative genomic data with the VISTA family of computational tools. Curr. Protoc. Bioinformatics.

[bb0340] Rebagliati M.R., Toyama R., Haffter P., Dawid I.B. (1998). cyclops encodes a nodal-related factor involved in midline signaling. Proc. Natl. Acad. Sci. U. S. A..

[bb0345] Reifers F., Böhli H., Walsh E.C., Crossley P.H., Stainier D.Y., Brand M. (1998). Fgf8 is mutated in zebrafish acerebellar (ace) mutants and is required for maintenance of midbrain-hindbrain boundary development and somitogenesis. Development.

[bb0350] Retnoaji B., Akiyama R., Matta T., Bessho Y., Matsui T. (2014). Retinoic acid controls proper head-to-trunk linkage in zebrafish by regulating an anteroposterior somitogenetic rate difference. Development.

[bb0355] Roest Crollius H., Weissenbach J. (2005). Fish genomics and biology. Genome Res..

[bb0360] Rovescalli A.C., Asoh S., Nirenberg M. (1996). Cloning and characterization of four murine homeobox genes. Proc. Natl. Acad. Sci. U. S. A..

[bb0365] Ryan J.F., Burton P.M., Mazza M.E., Kwong G.K., Mullikin J.C., Finnerty J.R. (2006). The cnidarian-bilaterian ancestor possessed at least 56 homeoboxes: evidence from the starlet sea anemone, *Nematostella vectensis*. Genome Biol..

[bb0370] Saito T., Lo L., Anderson D.J., Mikoshiba K. (1996). Identification of novel paired homeodomain protein related to *C. elegans* unc-4 as a potential downstream target of MASH1. Dev. Biol..

[bb0375] Sammeta N., Hardin D.L., McClintock T.S. (2010). Uncx regulates proliferation of neural progenitor cells and neuronal survival in the olfactory epithelium. Mol. Cell. Neurosci..

[bb0380] Sánchez R.S., Sánchez S.S. (2013). Characterization of pax1, pax9, and uncx sclerotomal genes during *Xenopus laevis* embryogenesis. Dev. Dyn..

[bb0385] Sawada A., Fritz A., Jiang Y.L., Yamamoto A., Yamasu K., Kuroiwa A., Saga Y., Takeda H. (2000). Zebrafish Mesp family genes, mesp-a and mesp-b are segmentally expressed in the presomitic mesoderm, and Mesp-b confers the anterior identity to the developing somites. Development.

[bb0390] Sawada A., Shinya M., Jiang Y.J., Kawakami A., Kuroiwa A., Takeda H. (2001). Fgf/MAPK signalling is a crucial positional cue in somite boundary formation. Development.

[bb0395] Schneider J., Skelton R.L., Von Stetina S.E., Middelkoop T.C., van Oudenaarden A., Korswagen H.C., Miller D.M. (2012). UNC-4 antagonizes Wnt signaling to regulate synaptic choice in the *C. elegans* motor circuit. Development.

[bb0400] Schrägle J., Huang R., Christ B., Pröls F. (2004). Control of the temporal and spatial Uncx4.1 expression in the paraxial mesoderm of avian embryos. Anat. Embryol..

[bb0405] Sewell W., Sparrow D.B., Smith A.J., Gonzalez D.M., Rappaport E.F., Dunwoodie S.L., Kusumi K. (2009). Cyclical expression of the Notch/Wnt regulator Nrarp requires modulation by Dll3 in somitogenesis. Dev. Biol..

[bb0410] Sinn R., Wittbrodt J. (2013). An eye on eye development. Mech. Dev..

[bb0415] Skuntz S., Mankoo B., Nguyen M.T., Hustert E., Nakayama A., Tournier-Lasserve E., Arnheiter H. (2009). Lack of the mesodermal homeodomain protein MEOX1 disrupts sclerotome polarity and leads to a remodeling of the craniocervical joints of the axial skeleton. Dev. Biol..

[bb0420] Stickney H.L., Barresi M.J., Devoto S.H. (2000). Somite development in zebrafish. Dev. Dyn..

[bb0425] Takahashi Y., Koizumi K., Takagi A., Kitajima S., Inoue T., Koseki H., Saga Y. (2000). Mesp2 initiates somite segmentation through the Notch signalling pathway. Nat. Genet..

[bb0430] Takahashi Y., Inoue T., Gossler A., Saga Y. (2003). Feedback loops comprising Dll1, Dll3 and Mesp2, and differential involvement of Psen1 are essential for rostrocaudal patterning of somites. Development.

[bb0435] Takahashi Y., Yasuhiko Y., Takahashi J., Takada S., Johnson R.L., Saga Y., Kanno J. (2013). Metameric pattern of intervertebral disc/vertebral body is generated independently of Mesp2/Ripply-mediated rostro-caudal patterning of somites in the mouse embryo. 2013. Dev. Biol..

[bb0440] Tamura K., Stecher G., Peterson D., Filipski A., Kumar S. (2013). MEGA6: molecular evolutionary genetics analysis version 6.0. Mol. Biol. Evol..

[bb0445] Taylor J.S., Van de Peer Y., Braasch I., Meyer A. (2001). Comparative genomics provides evidence for an ancient genome duplication event in fish. Philos. Trans. R. Soc. Lond. Ser. B Biol. Sci..

[bb0450] Taylor J.S., Braasch I., Frickey T., Meyer A., Van de Peer Y. (2003). Genome duplication, a trait shared by 22000 species of ray-finned fish. Genome Res..

[bb0455] Thompson J.D., Higgins D.G., Gibson T.J. (1994). CLUSTAL W: improving the sensitivity of progressive multiple sequence alignment through sequence weighting, position-specific gap penalties and weight matrix choice. Nucleic Acids Res..

[bb0460] Tyurina O.V., Guner B., Popova E., Feng J.C., Schier A.F., Kohtz J.D., Karlstrom R.O. (2005). Zebrafish Gli3 functions as both an activator and a repressor in Hedgehog signaling (2005). Dev. Biol..

[bb0465] Varga M.Z., Amores A., Lewis K.E., Postlethwait J.H., Eisen J.S., Westerfield M. (2001). Zebrafish smoothened functions in ventral neural tube specification and axon tract formation. Development.

[bb0470] Von Stetina E.S., Fox R.M., Watkins K.L., Starich T.A., Shaw J.E., Miller D.M. (2007). UNC-4 represses CEH-12/HB9 to specify synaptic inputs to VA motor neurons in C. elegans. Genes Dev..

[bb0475] Weinberg E.S., Allende M.L., Kelly C.S., Abdelhamid A., Andermann P., Doerre G., Grunwald D.J., Riggleman B. (1996). Developmental regulation of zebrafish MyoD in wild-type, no tail, and spadetail embryos. Development.

[bb0480] White J.G., Southgate E., Thomson J.N. (1992). Mutations in the *Caenorhabditis elegans* unc-4 gene alter the synaptic input to ventral cord motor neurons. Nature.

[bb0485] Whitfield T.T., Granato M., VanEeden F.J., Schach U., Brand M., Furutani-Seiki, Nusslein-Volhard C. (1996). Mutations affecting development of the zebrafish inner ear and lateral line. Development.

[bb0490] Windner S.E., Bird N.C., Patterson S.E., Doris R.A., Devoto S.H. (2012). Fss/Tbx6 is required for central dermomyotome cell fate in zebrafish. Biol. Open.

[bb0495] Winnier A.R., Meir J.Y., Ross J.M., Tavernarakis N., Driscoll M., Ishihara T., Katsura I., Miller D.M. (1999). UNC- 4/UNC-37-dependent repression of motor neuron-specific genes controls synaptic choice in *Caenorhabditis elegans*. Genes Dev..

[bb0500] Wolff C., Roy S., Ingham P.W. (2003). Multiple muscle cell identities induced by distinct levels and timing of hedgehog activity in the zebrafish embryo. Curr. Biol..

[bb0505] Woolfe A., Elgar G. (2007). Comparative genomics using Fugu reveals insights into regulatory subfunctionalization. Genome Biol..

[bb0510] Xue Y., Kuok C., Xiao A., Zhu Z., Lin S., Zhang B. (2010). Identification and expression analysis of mical family genes in zebrafish. J. Genet. Genomics.

[bb0515] Yabe T., Hoshijima K., Yamamoto T., Takada S. (2016). Quadruple zebrafish mutant reveals different roles of Mesp genes in somite segmentation between mouse and zebrafish. Development.

[bb0520] Zheng C., Karimzadegan S., Chiang V., Chalfie M. (2013). Histone methylation restrains the expression of subtype-specific genes during terminal neuronal differentiation in *Caenorhabditis elegans*. PLoS Genet..

