# Supplementary material for: Characterization of paralogous uncx transcription factor encoding genes in zebrafish
Source: Gene X. 2019 Mar 8;2:100011. doi: 10.1016/j.gene.2019.100011 (PMC6543554; doi:10.1016/j.gene.2019.100011)
Supplement: Supplementary file 1 — Supplemental File 1 FASTA file comprising protein sequences employed for Uncx phylogeny. Supplemental File 2 FASTA file including protein sequences excluded from Uncx phylogeny due to their molecular divergence. Supplemental File 3 Alignment of Uncx proteins showing the presence of a conserved intron/exon structure. Color-coded text highlights conserved splicing sites. Supplemental Fig. 1 Corresponding position of riboprobes. Red line: riboprobe. Red rectangle: exon. Orange rectangle: homeobox. White rectangle: untranslated sequence. Numbers indicate nucleotide sequence length. Supplemental Fig. 2 Synteny conservation of Uncx genes in Olfactores. Horizontal bars represent orthologous genomic regions of man (H. sapiens, Hsa7), ascidian (C. robusta, Cro11) and zebrafish (D. rerio, Dre3 and Dre1). Orthologous genes are shown with same colors. Lines highlight a conserved microsynteny among Uncx and Elfn1 genes. Supplemental Fig. 3 Syntenic conservation of Uncx genes in Metazoa. Horizontal lines represent orthologous genomic regions of annelid polychaete (C. teleta, Sc658: 20,810–22,694), insect (D. melanogaster, Chr X), hemichordate (S. kowalevskii, NW_003125889.1), amphioxus (B. floridae, NW_003101431.1), and human (H. sapiens, Hsa7). Orthologous genes are shown with same colors. Supplemental Fig. 4 VISTA plot of the intergenic space between Uncx and Micall2 genes in H. sapiens and D. rerio. Supplemental Fig. 5 VISTA analysis of Uncx gene loci in Metazoans. Exons are highlighted in violet and UTR regions in light blue, while conserved non-coding elements (CNE) are shown in pink. Horizontal lines showing peaks of similarity in pairwise sequence alignments between human Uncx locus vs. orthologous genomic regions of mollusk (C. gigas, Cg), brachiopod (L. anatina, La), ascidian (C. robusta, Cr), coelacanth (L. chalumnae, Lc), and actinopterygians (L. oculatus, Lo; D. rerio, Dr; T. nigroviridis, Tn). Orange and green boxes highlight lack of conserved regions upstream and in [file mmc1.docx]

**Supplemental files**

**Supplemental File 1**

>XP_002612717.1_Hox3_Branchiostoma_floridae

MAASFHPAPPRLVLSAAFRPTTTMARHRVFVLDTVLSHILWNPFRGPLDFAEDRPRPLSLGAIIGQVRSCDTGVPMLFDKSVKTLSQRNKFLQNNEMQKSPYYETTSQQLYNGYSYSNGERFGYEGSGGGGGGGSYGAEVTQDFGPSCSVRKPGSTTGGGTGNVSPTCMKTNPADNNHGSAPQSNASILANTKIYPWMKESRQNSKQRQQPNLSVGTTEPGESPGLGGAAGKRARTAYTSAQLVELEKEFHFNRYLCRPRRVEMAAMLNLTERQIKIWFQNRRMKYKKEQKVKGGGSGGGSGGMNSPSPPATTTPPGINPGPLPHPTTQPSLSQSNNMSNHMSMMGSLQQTQPAYSQYPPPHLNHSLPHQAPPHSVGLTMSGPVSPQCYQSNHSPCPPSSAPHPVPMGNHVPHPRQGPPHMTNGLPASPLEVVSQRQYTPPAPGPSPTGPGPGHHGLTNSYPECPPHHTELPHHQYSTPMSVVNMNYCVNVQTQGNRLNSAPKLTHL

>XP_019632164.1_Hox3_Branchiostoma_belcheri

MLFDKSVKTLSQRNKFLQNNEMQKSPYYETTSQQLYNGYSYSNGERYGYEGSGGGGGGSYGTEATQEFGPSCSVRKPASGPGSGPGNVSPTCMKTNPADNNHGTAPQSVSNAAILANTKIYPWMKESRQNSKQRQPTNLSVGSTTEAGESPGLGGAAGKRARTAYTSAQLVELEKEFHFNRYLCRPRRVEMAAMLNLTERQIKIWFQNRRMKYKKEQKVKGGGSGGGSGGMNSPSPPVTTTPPGVNPGPLPHPTTQPSLSQGNNMSNHMSMMGSLQQTQPSYSQYPPPHLNHSLPHQAPPHSVGLTMSGPVSPQCYQSNHSPCPPTSAPHPVPMGNHVPHPRQGPPHMTNGLPASPLEVVNQRQYTPPAPGPSPTGPGPGHHGLTNSYPECPPHHTELPHHQYATPMSVVNMNYCVNVQTQGNRLNSAPKLTHL

>ENSTNIP00000021778.1_Uncx_Tetraodon_nigroviridis

MMDSRILDPPHAQFGSSLGGMVGFPYHLSHHHVYELTGHQLQSASAVPFSIDGLLNGSCAASVGNSNPLLSSGCGMNGDNQHYKLTDSGDPDKDSPGCKRRRTRTNFTGWQLEELEKAFNESHYPDVFMREALALRLDLIESRVQVWFQNRRAKWRKKENTKKGPGRPAHNAHPTTCSGEPMAPEEIARRELERLEKKKRKQERRLLKSQNKFQSGDLLHTPGSDSDTGVSHVTDSDHIPCQPFDSVGGNQSCDQTPHSFQTAEQNQRHLDQDAGAPELDSPDAGHRSSLCSGSSRASGAQKLNPFSVESLLSDSRPRRNLAALPSSRPLIGKGHFLLYPITQPLGFIVPQTALK

>ENSTNIP00000011069.1_Uncx4.1_Tetraodon_nigroviridis

SMDSRVLEHPHAQFGGSLGGLVGFPYPLAVYELASGNQLQSAAAVPFSIDGLLSGSCSASVVAATPLLTPDSQFKLSDSGDPDKDSPGCKRRRTRTNFTGWQLEELEKAFNESHYPDVFMREALALRLDLVESRVQVWFQNRRAKWRKKENTKKGPGRPAHNSHPTTCSGEPMDPEEIARREQERAEKKKRKQERKLLKSQGKLLPGESFHTPGGSESDSGVSQFTDSEQQSSNHNGTTPTELPSAKRAAGHQTDSSCDQTRHNFSQLQNHPNQRTAGEPEQISPEGTHSSGPAGPRCSTLQKRNPFSVESLLSDATPRRKSQLDFSSARTLVGKGHFLLYPITHQPLGFLVPQTALKASPGPD

>XP_005164261.1_Uncx_Danio_rerio

MMDSRILDPPHAQFGGSLGGMVSFPYHLSHHHVYELAGHQLQSTAAVPFSIDGLLNGSCTASVVNSNPLLSSGCGMNGDNQQYKLTDSGDPDKDSPGCKRRRTRTNFTGWQLEELEKAFNESHYPDVFMREALALRLDLIESRVQVWFQNRRAKWRKKENTKKGPGRPAHNSHPTTCSGEPMDPEEIARRELERLEKKKRKQERRLLKSQNKLLPGDLFHTPGSDSDSGLSQITDSEQSLHCDMGRNQTQPSCDQTPQKLQNQRNADQDASGSELDSSDSGQQSNLCSNSRSSALQKLNPFSVESLLSDSRPRRKPPMDFPVPTPRPLIGKGHFLLYPITQPLGFIVPQTALKSTAPGPDTENGQKGPATDITFAGNPGHRNAKENNSVNNNSGARAIKGQVSQSGNISCSTQSSSPQTTINGHSAGSCNERCPQDIELELVDPKSPQSEKKEQSLPDYPPQNSESATTSKDTDKDSVDVDME

>NP_001018616_Uncx4.1_Danio_rerio

MMDSRILEHPHAQFGGSLGGMVGMGFPYHLGHHHVYDISGHQLQSAAAVPFSIDGLLNGSCSGSVANSNPLLGSGCGVNVDSQYKLGDGGDPDKESPGCKRRRTRTNFTGWQLEELEKAFNESHYPDVFMREALALRLDLVESRVQVWFQNRRAKWRKKENTKKGPGRPAHNSHPTTCSGEPMDPEEIARRELERLEKKKRKQERKLLKSQNKLLAGELFHTPGSDSDSGVSQSTDSESTPHTGPQHSAHRQQTEHICEQHARHQRASTVNETAEPMDSTRNSGLCPANGITRASTLQKLNPFSVESLLADSSPRRKTILDFSQLPPQRPLVGKGHFLLYPITQPLGFIVPQTAMKQSHDSGNSGHHCSTTDTSTSNQKNVNHLCRDNTGASDELQRETKNSSIQSPSTSSEKCFSESNSPQKESENDSESTVTNSSQKESISANLSEYSDRKSRSSADTNTDGEDVDMD

>NP_001073930.1_UNCX_Homo_sapiens

MMDGRLLEHPHAQFGGSLGGVVGFPYPLGHHHVYELAGHQLQSAAAAASVPFSIDGLLGGSCAAAASVVNPTPLLPAACGVGGDGQPFKLSDSGDPDKESPGCKRRRTRTNFTGWQLEELEKAFNESHYPDVFMREALALRLDLVESRVQVWFQNRRAKWRKKENTKKGPGRPAHNSHPTTCSGEPMDPEEIARKELEKMEKKKRKHEKKLLKSQGRHLHSPGGLSLHSAPSSDSDSGGGGLSPEPPEPPPPAAKGPGAHASGAAGTAPAPPGEPPAPGTCDPAFYPSQRSGAGPQPRPGRPADKDAASCGPGAAVAAVERGAAGLPKASPFSVESLLSDSPPRRKAASNAAAAAAAGLDFAPGLPCAPRTLIGKGHFLLYPITQPLGFLVPQAALKGGAGLEPAPKDAPPAPAVPPAPPAQASFGAFSGPGGAPDSAFARRSPDAVASPGAPAPAPAPFRDLASAAATEGGGGDCADAGTAGPAPPPPAPSPRPGPRPPSPAEEPATCGVPEPGAAAGPSPPEGEELDMD

>NP_038730.1_Uncx_Mus_musculus

MMDGRLLEHPHAQFGGSLGGVVGFPYPLGHHHVYELAGHQLQSAAAAAAAASVPFSIDGLLSGSCAAAAASVVNPTPLLPAACGVAGESQPFKLADSGDPDKESPGCKRRRTRTNFTGWQLEELEKAFNESHYPDVFMREALALRLDLVESRVQVWFQNRRAKWRKKENTKKGPGRPAHNSHPTTCSGEPMDPEEIARKELEKMEKKKRKHEKKLLKSQSRHLHSPGGLSLHSAPSSDSDSGGGGLSPEPPEPPPPTAAAKGPGAHGSGIAGSAPVPPGEPPAPGTCDPAFYPSQRSGAGSQPRLGRPADKDTVPCGPGAAATAGLPKASPFSVESLLSDSPPRRKATPANAAATAGLDFTPGLPCAPRTLIGKGHFLLYPITQPLGFLVPQAALKGGAGPELVPKDAPPAPPAPPAPPAQASFGTFPGPGGAADPAFARRSPEVVASPGPPAPASFRDLTAAAAESGAGDCADVGTVCPAASPPPPLETSPGPGPRAPSPPGEPATCGAAEPGAATGPSPPEGEEVDMD

>ENSLACP00000011216.1_Uncx_Latimeria_chalumnae

MMDSRILEHPHAQFGGSLSGMVGFPYPLGHHHVYELAGHQLQSATAVPFSIDGLLNSSCTASVVNPNPLIPSGCGMNGDNQQFKLTDSDPDKESPGCKRRRTRTNFTGWQLEELEKAFNESHYPDVFMREALALRLDLVESRVQVWFQNRRAKWRKKENTKKGPGRPAHNSHPTTCSGEPMDPEEIARKELERMEKKKRKHERKFLRNQSKHLHSPSYSIHTNGSDSDSGLSQLQDSGQSEPDPGQIRDTPETRNQTEPNCDQNGQNMFQSQRNPGQQAGGSENDSSDSNNSNLCTSGRSSSLQKLNPFSVESLLSDSTPRRKPVLDFAGLSTPRPLIGKGHFLLYPITQPLGFIVPQTAIKTTSSPEASNLVQKDLSTNQGQSSFTTTKTGQHSYPGTTNKGHGTFPGKNASSIPTNYSSSNPGDSNCPGPNSTSTQPQASYLESRSQHADCQQPEPKSPVSNSKDNVTKNKSDCVEQSKTNCPPDTSTDCEEVDMD

>ENSLOCP00000001388.1_Uncx_Lepisosteus_oculatus

MMDSRILEHPHAQFGGSLSGMVGFPYHLSHHHVYELAGHQLQSAAAVPFSIDGLLNGSCTASVVNSNPLLSSGCGVNGDSQQYKLTDSGDPDKDSPGCKRRRTRTNFTGWQLEELEKAFNESHYPDVFMREALALRLDLVESRVQVWFQNRRAKWRKKENTKKGPGRPAHNSHPTSCSGEPMDPEEIARRELERMEKKKRKQERKLLRSQNKLLSGDLFHTPGSDSDSGVSQITDSVDSA

SNMAPSESSGRNQTEQSCDQTRQKLQSQRNLGQDAGGSEQDSPDSNHNSNLCSNSRASALQKLNPFSVESLLSDSTPRRKTTLDFPALSTPRPLIGKGHFLLYPITQPLGFIVPQTALKAAPSPDSVSSGQRSAAADCSSAQPSLANTSPGHSKSKTSSTTNSSSPTNNEQSSFTGQTDSSPQSNLVPTTYSRRSSAQSPGCYSEPHPQLSDSTQDQPESPHSDTKENSAPDHSECSETAKTNCPPDTNTDCEDVDMD

>NP_001305673.1_Uncx_Xenopus_tropicalis

MMDSRILEHPHAQFGGSMSGMVGFPYPLGHHHVYELASHQLQSAAAAVPFSIDGLLNGSCTASVINPTPLLPSGCGLNGDSQQYKLSDSIDPDKESPGCKRRRTRTNFTGWQLEELEKAFNESHYPDVFMREALALRLDLVESRVQVWFQNRRAKWRKKENTKKGPGRPAHNSHPTTCSGEPMDPEEIARKELEKMEKKKRKQEKKMLRNQNRLQHSPGDMSLHTPSSDSDSGLSQNLDSSSLESSHPDMGQSRSQTQASCDQTGQAFFQSQRNTGQRDRVSDKESPVEANHNSGLCPNGRGAAFQKLNPFSVESLLSDSPPKRKTGIEFPGLTNPRTLIGKGHFLLYPITQPLGFIVPQASIKSNSAQELPSPGIKDSLHNSNSGQPSLNTSPNSAQNLYPNKHTNSTQSTTLTVCKTENGDHHGATSSSGDTGTEQLDSTYSEPKSPKSLKSDRETPECPNGSTSTCPPKPSLDCEEVDMD

>XP_007903911.1_Uncx_Callorinchus_milii

MREALALRLDLVESRVQVWFQNRRAKWRKKENTKKGPGRPAHNSHPTTCSGEPMDPDEIARREMEKLEKKKRKQEKKLLKSQSKLLHGDQAGDYSLMSSGSESDSSQMKFPQCRELTIRAEPGQQRSPSPSPGQRDPSQGDCEPPSGPKPPPPHQRNGDGGPPPLQSDAHPSDADCEAENPLHDHSHHSCPNRSASNLPKCEQKTNPFSVESLLSDSTPRRKVHFDFPTLPNPRAVIGKGHFLLYPITQPLGFIVPQSSIRTSPAQLTQPNQPPTSSAAATTTLPPASPATEPAHHQHHHHNTAYPGTHSNSTQTACPGTDSSPAQPATPPQPAPVPAQAQAPAPAPAQAQATSGYPQPNPNQPDPQYPEPNQKPVVADRQGELSRDKPDSPEPVKASCPGEHMTECEVVNMD

>NP_496138.1_Uncx_Caenorhabditis_elegans

MIGALHACVDAEPKIINDIWADFWKSQINSVLLNPSDGSETYLASDNGKSTSSREQSTSPDDDNLLMNEDDGIALEDDNDTGESAAKRRRTRTNFSGWQLEELESAFEASHYPDVFMREALAMRLDLLESRVQVWFQNRRAKWRKREQNRNGSSEIKKDDGEQMETKALPTFPFSIDSILAVSRVPRGRRPNAKYPRVQACKNLSPFMIPLFPITQPGGNVIREKSPPLPTQQSQIVATNALTTVAELLKSV

>XP_011445596.1_Uncx_Crassostrea_gigas

MLYYCDRGRKGRNKMSAGPLGLLDARYAHGYGLLSSLSGYAAHFGGPFASLGFGNPYGIDFGSHQVGHGAYMEGLMSASSPTSRNSNTHPLGSPSAKSSVKSPDSSDEDRESAAAKRRRTRTNFTGWQLEELERAFQDSHYPDVFMREALALRLDLVESRVQVWFQNRRAKWRKKENTKKGPGRPAHNAHPQTCSGEPMDPEEIKRREQEKMEKKKRKQEERLRKLEERRKQMQDQKQGPYSRLSDSDTSPSKADSCNMSVSSCNDNSNHGDLPDQPEDEEDAVSDQENRKCSFSIDSLLEASKVPRGRRPNSKYPRVQASKSVNALGLGMMPLFPITQPVGFIVEQRSHASLSDDEMSINSDDESLHENNSSNEDSHINVEQFSDVEDEAQKSPAPCPKSPDPCS

>BAQ19204.1_Uncx_Lottia_gigantea

MSAYPHFVSPFSALRAPSPEQLRALDCTVNPNFYRHGQMNFSMDKIITSMKDGRASPESSDGNSDCESKFNGRDDDRESAAAKRRRTRTNFTGWQLEELEKAFNDSHYPDVFMREALALKLDLVESRVQVWFQNRRAKWRKKENTKKGPGRPAHNAHPHTCSGNPMDAEEVRRREEERLLRKQRKQEERLRRLEEKRNTFGNKQSLNITSRLEMMSKSGSESDKSNDMFTNNESHRQHIAGVENNSKSPYSIASLLEKSKVPRGRRPNSKYPRVQACKSLGPIGIGMMPLFPITQPIGFVVEQLPESDEEDMEDDLDVVSVENEEQNKPSSTEDVEHSDSNEALDFSKSTAQESVSGSEEQ

>XP_002595577.1_Uncx_Branchiostoma_floridae

MMDNCLITQQFPHLNGVNLPGFPYSHPPYPHAFDFPPTSSGLPGPRPFSIDGILSGGGHPSVSFPQTSMMLNGGINVITTTAGNNKQESNNLRDSLLFSFVENGQDSGEGMPPKRRRTRTNFNSWQLEELERAFQESHYPDVFMREALALRLDLVESRVQVWFQNRRAKWRKKENTKKGPGRPAHNAHPQTCSGDPIPAEELERRQREREERKKRKQMEKQRQKGSIRADGSQSGGEDGMGKSGLSGDLDSSTDNPCRADGESRWGESDSGNTTDSSSASSRYDQVRAREDVTKDLAPASSGDGEKEHKDDTAQHESAETHSSEQFNNSTEEVVITEENMSASETTLNRDSVSVSDTTTTTCPAVTTTPPCTTPSPTRPATSPLNTVTKSPYSIESLLAEPKVPRGRRPNTKYPRVQASKSAYSNGLVPVYPITQPVGFMVQPSVATAVSPTQVSPTLPLAAERQNSSIAALRLKAKEHQITIDSQLENFRCSPDRCSV

>XP_019642617.1_Uncx_Branchiostoma_belcheri

MMDNCLITQQFPHLNGVNLPGFPYTHPPYPHAAFDFPPTSSGLPGGPRPFSIDGILGGGGHPNASFPQTSMILNGGINVITTTAGNNKQENGQDSGEGMPPKRRRTRTNFNSWQLEELERAFQESHYPDVFMREALALRLDLVESRVQVWFQNRRAKWRKKENTKKGPGRPAHNAHPQTCSGDPIPAEELERRQKEREERKKRKQLEKQRQKGAMRAEGSQAGDDGAEKASLSGDLDTSTDNPCRADGESRWGESDSGNTTDSSSTSSNYERVRARKDDTTAASLVVTDDQEQHHQDDTSSHQTPSHTEHFNTTEEAAATTEENLPSSETTLDRDSVSVADSATTTCSTVSTTPPCTAQSPTRPATSPINTVTKSPYSIESLLAEPKVPRGRRPNTKYPRVQASKSAYSNGLVPVYPITQPVGFMVQPSVATAVSPTQVSPTLPLAAERQNSSIAALRLKAKEHQITIDSQLAENFRLSPDRCSV

>XP_002734968_Uncx1_Saccoglossus_kowalevskii

MDSRLLDQHYGRSLGSLAAAFPYRPGHAFEFPSVHNLSNSVSFSIDGILSTNSLINGSSTGSQMLGTTTITSTNNSNIHITSNDSEHDGKESSSGKRRRTRTNFNGWQLEELEKAFNDSHYPDVFTREALAMKLDLVESRVQVWFQNRRAKWRKKENTKKGPGRPAHNAHPTTCSGVPIDPEELKKKEHERTERKRRKQEDKARLKALKEATKNNKDLDIKGLESLKSLDLSSSKMDFDSCKSTASESYISERDTEDRNHKHEDDILQRIAKEVNDANRNHDLKENNDTRLHRGDTVGRTGHENDGTIVAELSTKKSPSGQQKSAFRSPFSIEQILSSDSSTRETKLKNNKIQRLKSSVETSVSVGLYSVYGVAQPIGLLVKGATTASSPPLPSPVSPIVTNLKGSNVSEDRQSSSIASLRIRAQEHKYAMKTELEVRNLVC

>XP_782932.2_Uncx_Strongylocentrotus_purpuratus

MDAQLMRSHFSQGSLGGLSPFPAYSFSSAAHALGFHVSPPGASPGTHGDLAITTSMAAAAAAVRVQSSMKKDLKHVSGDCFSSKSKMMKIGGIEDDHDKNDKDDKDSNDNKRRRSRTNFNGWQLEELERAFNESHYPDVFTREALAMRLDLVESRVQVWFQNRRAKWRKRENTRKGPGRPAHNAHLTSCSGDPIPPDELERRERMKMERKIKKQKEKTDKVPNRQVSRRVGGGSQAEGSGGGDGEERRRDSCSSSTGICVDGPGGFLADGHLSQKDFMESSSAEPSEIDSECMDSMSIDTTEEQRKLPDSPRFKLAARTLLNPLRGVGPLPRFDGDKLQDCDAASAFHSSFSIDRLLSRQPEQHRDIKPGTVAAAAAAAALWPDFCPSYALAAPNPLAWGALTTFGFPRMWAGLTADRLAGCLRANSQQMGLLDAWRERKTMSVEALRKKAQEHKEALSKNELSDG

>ELT92834.1_Uncx1_Capitella_teleta

RTRTNFTGWQLEELERAFQDSHYPDVFMREALALKLDLVESRIQVWFQNRRAKWRKKENTKKGPGRPAHNAHPKTCSGDPIPEDELRKRDQERMEKKRRKQEER

>ELT92833.1_Uncx2_Capitella_teleta

MFFKTPLSKSSFYVLTEISDEDKRRRTRTNFTGWQLEELERAFHDSHYPDIFMREAIALKLDLVESRVQVWFQNRRAKWRKKENTKKSPGRPAHNAHPLTCSGDPISEDELRRREEQKLLRRMRKKVEEVRVPSTPLQSCSSFSIDSLLNHTPRRPQVQRPRPNALSFTQSLGFPVERLPTPPPEESSSSPVMSVGTKSFAVFPEPEVAIVDNGVQSR

>XP_015149815.1_Uncx_Gallus_gallus

MMDSRILEHPHAQFGGMVGFPYPLGHHHVYELAGHQLQSAAASVPFSIDGLLSGSCAASVVTPAPLIPSGCGVSADTQPFKLADSGDPDKESPGCKRRRTRTNFTGWQLEELEKAFNESHYPDVFMREALALRLDLVESRVQVWFQNRRAKWRKKENTKKGPGRPAHNSHPTTCSGEPMDPEEIARKELEKMEKKKRKHEKKLLKSQSRHLHSPSGLSLHSTPSSDSDSGGGGGLSPAPPEAKPRGQPPPPHPPPPAAAASSEPPPSSCDQTAEPFYPSQRSGAGRLQRPPDKDCAAAPSGDGSGGAGGTRGAGSFHKLNPFSVESLLSDSPPRRKAALDFPSLQPCAPRTLIGKGHFLLYPITQPLGFIVPQTALKANPGPEAGQPPRDSPLPAANPGPPAFSAEPAPAGAQPGPSSTDTAAGPAPPPPAGASPRSAAAHGDPAAAAPAEGGSFPRPKSPVRARDGSATPRDKSDCGPAEVGGLDGEEVDMD

>g19407.t1_Uncx_Lingula_anatina

MLSYFPNAAAVSSVTGKMENRVFGNSPAAHLGRGIGFTPFSQHSYPTPLPACGPYGYEFPGGNLGHSSFSMESLLPRGQGSPTSLTPGSRISSHHSPDPGSDLKDGEKDDCHPKRRRTRTNFTGWQLEELERAFQDSHYPDVFMREALALRLDLVESRVQVWFQNRRAKWRKREHTKKGPGRPAHNAHPQTCSGEPIDPEEVQRREQERLEKKRRKQEERKKRIEEKKRVLGSSKCGLSRDSDVLLSQSQDVDSRIDDSEDAIDVVGDNSDGDASRASEEPSSHDENSNRVLTFRSAFSIDCLLEAPKVPRGRRPNSKYPRVQASKSMNPFSLGMVPLYPINQPVGFMVEQIPENEESERLSEARSPQNVRVEPETHNGTEPENNEFAVLDYSVVSEPDSEMPQKQNQ

>XP_022092302.1_Uncx_Acanthaster_planci

MDSPIVGGQLPHSLGPLGLYAFGSPGLGFPSSRAFDFGLPVSSASTGAFSPDGFLAKGSQLSDVSSCGSSVSKAKASPGGESMLDGEGKDSNGSKRRRSRTNFNGWQLEELERAFNESHYPDIFTREALAMRLDLVESRVQVWFQNRRAKWRKKENTKKGPGRPAHNAQLVTCSGDPIPPEEIERRERLRLEKKKQKQREKAEKNLAKAKKQGGAAEATGKKDAIAAEGGSDDTERGDEDPEDRDSKCSLPLLSPGTNSTQETTTDVFLPEKKLKTPGGKLPKGDKTRREGKQRSKLPHHCGVDSANSAFRSSFSIECLLSPRPSDVGGRGFGLLAGHYAAAASLAASPYAAAFSPFTWNSLSAALAAGVPQPLGYLVERLLPSKPHNLPIMAHPRPLTDVFEEQKTLSVERLRKKAEEHMSKVAVQKESSSDTESTKMELS

>XP_015283125.1_Uncx_Gekko_japonicus

MMDSRILEHPHAQFGGMVGFPYPIGHHHVYELAGHQLQAAAAAAASVPFSIDGLLNGSCAASVVNPTPLLPSGCGDSQPFKLSDAGDPDKESPGCKRRRTRTNFTGWQLEELEKAFNESHYPDVFMREALALRLDLVESRVQVWFQNRRAKWRKKENTKKGPGRPAHNSHPTTCSGEPMDPEEIARKELEKMAKKKRKHEKKLLKSQARNLHSPSGLSVNSSTQSSDSDGGGGLSPDPPDGCKASLGGPPNPPLPGSCDPTGPAFYPANRGGGGCSSTSCPAKEASPSGSDCPAPPSSTARPPGGFPKLNPFSVESLLSDSPPRRKAPLDFPPALAPCAPTARTLIGKGHFLLYPITQPLGFIVPQTALKGHPGQEPPQRGSPPPAANPSPDPAPPKSSTTAAISSTPGSEPAGALGPRKAAPSPPAPATASSSSSPPSYCDSSSPQADSSGAAAAQPKSPLLLHPPAKDACQSRDNSDCRAEPGCPEETSNLDCEEVDME

**Supplemental File 2**

>ENSEBUP00000013593.1_Uncx_Eptatretus_burgeri

GMERRLLDYQATPFGLGLPTLTAFRYPLTPHPLYDVPSQRLEARPGPAPFTIDGLLSAACPEPFGPATTFLSGDTTLSITGKENPGCKRRRTRTNFSGWQLEELERAFNESHYPDVFMREALALRLDLVESRVQVWFQNRRAKWRKKENTKKGPGRPAHNAHPVTCSGEPMDPTDIARRE

HERAEKRKKKELRLQNCQKEGDIRLAIDDSDSSADRSITASPQSVKTTQVDAVVAAVTTVAPVSEPEGAEAKDATTRCAVEKLNMKQILHPKSNPFSVESLLAERPRNTRRDFLFPTLACSAASPKASSTVGEKGLTFYPVTQPLGFMVQQQQQPDLPPLVPVTDTPPRDEAPSKGSAATEPSPEPESSRDSPAMPTSAHADPVQGHEPQERGSQARTCLTTSPEPQGTWDGRVGSSATVIGTETVTSSERKEPSEGVCERGM

>ENSTRUP00000040497.2_Uncx4.1_Takifugu_rubripes

MDSRVLEHPHAQFGGSLGGLVGFPYPLAPHHHHHHHHQHVYELASGNQLQSATAVPFSIDGLLNGSCSASVVNSNPLLSPDSQQFKLSDSGDLDKDSPGCKRRRTRTNFTGWQLEELEKAFNESHYPDVFMREALALRLDLVESRVQVWFQNRRAKWRKKENTKKGPGRPAHNSHPTTCSGEPMDPEEIARREQERAEKKKRKQERKLLKSQGKLLPGDSFHTPGGSESDSGVSQFTDSEQQTSSHNGTTHIELPTAKRAAGHQTESSCDQTRHNFSQLQNHQNQRTPGEPEQISPESTHSSSPGGPRSSTLQKRNPFSVESLLSDATPRRKSQLDFSSSRTLVGKGHFLLYPITHQPLGFLVPQTALKASPCQDSSISRLGQRCESAEGSPTSSDLSGFIPKLLNSDNVDSGSARRHREHISNRPDGADAEDCEDGGGGERLPVSPLSNTEAGQARLISIPAADSEDEKSSRAANNGYQELRAKEERASPECLETDCQEAPATDGEDVDMD

>ENSTRUP00000034443.2_Uncx_Takifugu_rubripes

MMDSRILDPPHAQFGGSLGGMVGFPYHLSHHHVYELTGHQLQSASAVPFSIDGLLNGSCAASVGNSNPLLSSGCGMNGDNQHYKLTDSGDPDKDSPGCKRRRTRTNFTGWQLEELEKAFNESHYPDVFMREALALRLDLIESRVQVWFQNRRAKWRKKENTKKGPGRPAHNAHPTTCSGEPMAPEEIARRELERQEKKKRKQERRLLKSQNRLQSGDLLHTPGSDSDSGVSHVTDSDHIPCQPFDSVGGNQSCDQTPHSFQTADHNQRHLDQDADAPELDSSDAGHRSSLCSGSSRTSGAQKLNPFSVESLLSDSRPRRNLAALPASRPLIGKGHFLLYPITQPLGFIVPQTALKPTAAAAAAANPDSDPPAGRSHAGVSARSAGCRSGSPDSADASAQSGLVTPAEQARGSPPRAAPGAGKSTHISVICGDSTFAHEHGAQVIQPVDADRKGHSEGKEQPVSTCAENNADSKEDVDLE

>AAW23068.1_Uncx_Oikopleura_dioica

MNPWRALELSRIFALNALGTPTTYDYGQQQREILSNVLAYQQRALAQRGSTRSTQDNSSSTSSSTDSENNKDPLPLIPLCLPPPDHLIEESKRRRTRTNFTQLQINELEKAFNQSHYPDIYMREALALRLELAESRVQVWFQNRRAKWRKRENTRKSPGRPPQGAHLLSCSGEPLSPEEIEARELRKKRKQARINFEVFDDNSSIPPNFTMDFILRKADHSEEREVLTPKSEQNSAQSS

>XP_009860233.1_Uncx2_Ciona_robusta

MYAPQPLVPSSMMTYPPPLYHRPSARFSLSPSAGNGGSGCPKNYYQHHVTNHAAAAAAAMYAGGAYDGFLQNYVPTDPCKQALMMASQAAGFTGMGNDYNACLDDSKPTKQRRARANYSQWQLEELERAFHTTHYPDIFMREALALRLDLIEARIQVWFQNRRAKLRRQLKMQNKTNKNDAEKNKDENSDESKSENSSVTEDVCKKGEAPVFDDVNGQGETKQDKKKR

>XP_009858714.1_Uncx1_Ciona_robusta

MNMFLSNPFLQMCYPSAISGQIKPETQVPPGFIKNNQTSPPYSLPYLFHSSSTESDKMSSSATECFSPVFTRRSTDTSYKTEMTSDASLSHQLPSDLWARNSFLSQCILQNSKEANSTDFYDDDSAENQQNKRRRTRTNFSTWQLEELEKAFGNSHYPDVYMREALALRLDLVESRVQVWFQNRRAKWRKREHTKKGPGRPPHNAQLTLCSGEPMDPSEIERREKMKQERKKRKQDCNCKSSLNEKQGRKTSNNQDSISPHNDSCKDRNIIQKTCCDSLNAKNNILLPWLVNQPPQSINHELKISENETLGSPLSIENDNQMTDGRKEMSDANPMTPGSRNSHSIENILYKNKIAKPQFFNHVPGTSGTLTSPSSSFGNLPMQSSPLFAEFIRRSSIFSLFPNIPKELQESTAADLNNYTRLYQQYQLQLRVFSDSFSNALNKGKQ

>XP_002734969.2_Uncx2_Saccoglossus_kowalevskii MWELQYHGKTIKMNPAIFGSPPYTPPLTANFQSFAASNAFGLPIGTLANAARCSADFDILAAAAATSALYSPGPMCPYQSMLSPNYVPSEPCRQALMMASQAAGLTGATEYSAEENKPIKQRRNRANYSAWQLEELEKAFQTTHYPDIFMREALALKLDLIEARVQVWFQNRRAKERRQMKIQGKDVTSKTGPSKIPRPLKEACSEKTTTPKEFPWPGSRSDGEKPHLAPPFVDILKASDNCSKLEERINPLTIESDDRRNSSIATLRQKAKLYMSELETHNNRDVIGSFSVENLSKEITNVAENKDDRNRTSPIVVVC

>NP_001285389.1_Uncx1_Drosophila_melanogaster

MVLETSEGDTKKFLTNNNNSSASRNNNSHNNNNNNHSPKEIPEETGRSSSTSSNSIPNAHRTNAGQHLLGGSPSSACSTSVSGCGMPSEGLHPTAALQLYAAAAQLAPNGVRVPPWGPFLQFGVPGVFGPNGPFLGRPRFDAASAGGHPNSAAAAAAATQMAAVNASNAFANLTGLSAAALRNVSAAQTTAVAAVASTVATIQHRLMIGNRQSLPPAGPPSEGSNEDGGFPGDGDDDSSAAKRRRSRTNFNSWQLEELERAFSASHYPDIFMREALAMRLDLKESRVAVWFQNRRAKVRKREHTKKGPGRPAHNAQPQTCSGEPIPPNELKAKERARRRKKLAKAIDRQARKLQAKGITVDLEALKAEYISQHKANGTFSDSDLEDDGIQIDVVGGTDSDDEGDSDAVSPVRLGGGGGGGGGTGGGGGGGASSSLHCGLDGDGDSSRAGSFIGGGGSLGSPSSVAPPSMLSNGAVQFGKLEPMDGNESEERDRERDSPKPLLFPAKAFHQLNLQSSQQHHGLVVGQGQGSGSGHGHGHQHNHHQHHLHHGSASASAAAAVANLVHQTSPISMRRSNPFSIESLLFNNT

>NP_523389.3_Uncx2_Drosophila_melanogaster

MDQLIQQLQQAAAARGQVQHPHGSSALQLYAAAAAVNMQVSGWSSVLNLSMDAPNPEISPNSVTNSSSVYMIRQMALIQQARVAAAAVAMQQQQQQQRDLNRELGMDPHSEQRIKLDSVSPTHNIHAGSSRGIKQDPLSDEGADSNLGQNDCTESSKKRRGRTNFNSWQLRELERVFQGSHYPDIFMREALATKLDLMEGRIAVWFQNRRAKWRKQEHTKKGPGRPAHNAHPQSCSGDPIPLSELRARELAQRSKRMKKAIDRQAKKLQDKGLEVDYARLEAEYLAAHQENGVDENNWLDDDGYDDLHIDVVGVEPEYVTGDSLDHSFCSSRTYQTKSTSSELDSNDMGL

QGRVETPPPPQPPMQNKTLYNSPFSIESLLGS

>ABB72463.1_Uncx_Nematostella_vectensis

RMRVRTNFSPWQLEELEHAFETTHYPDVFMREALAMRLDLTEARVQVWFQNRRAKWRKRE

**Supplemental File 3**


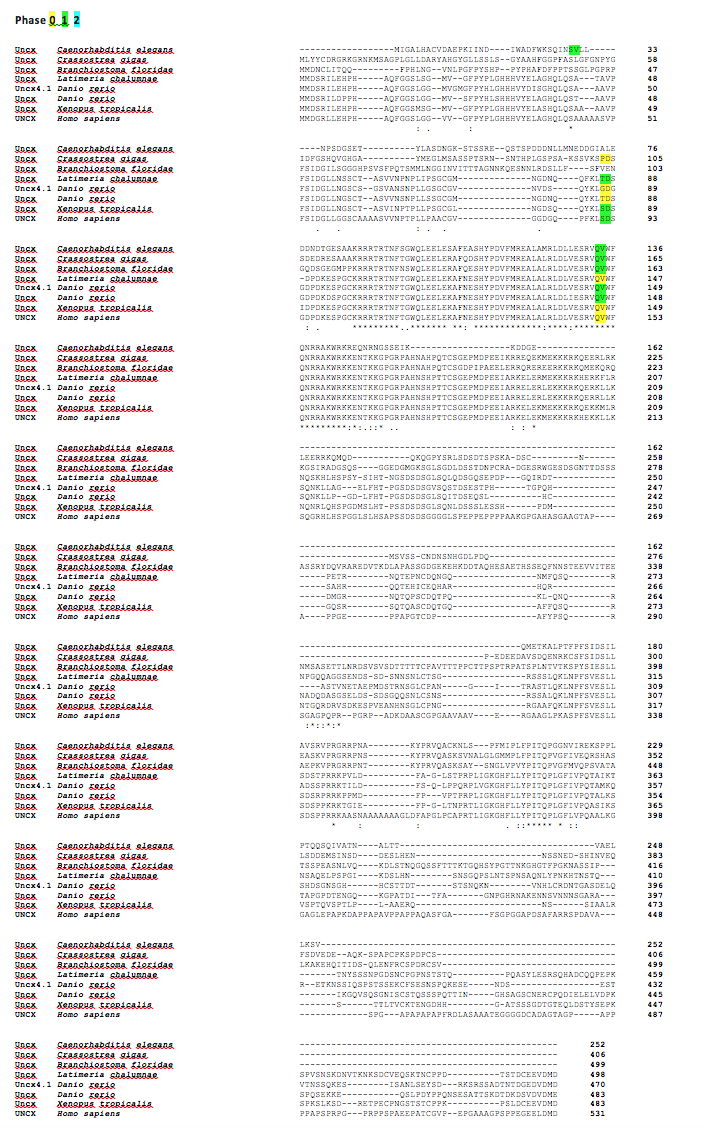


**Supplemental Figure 1**

**Supplemental Figure 2**


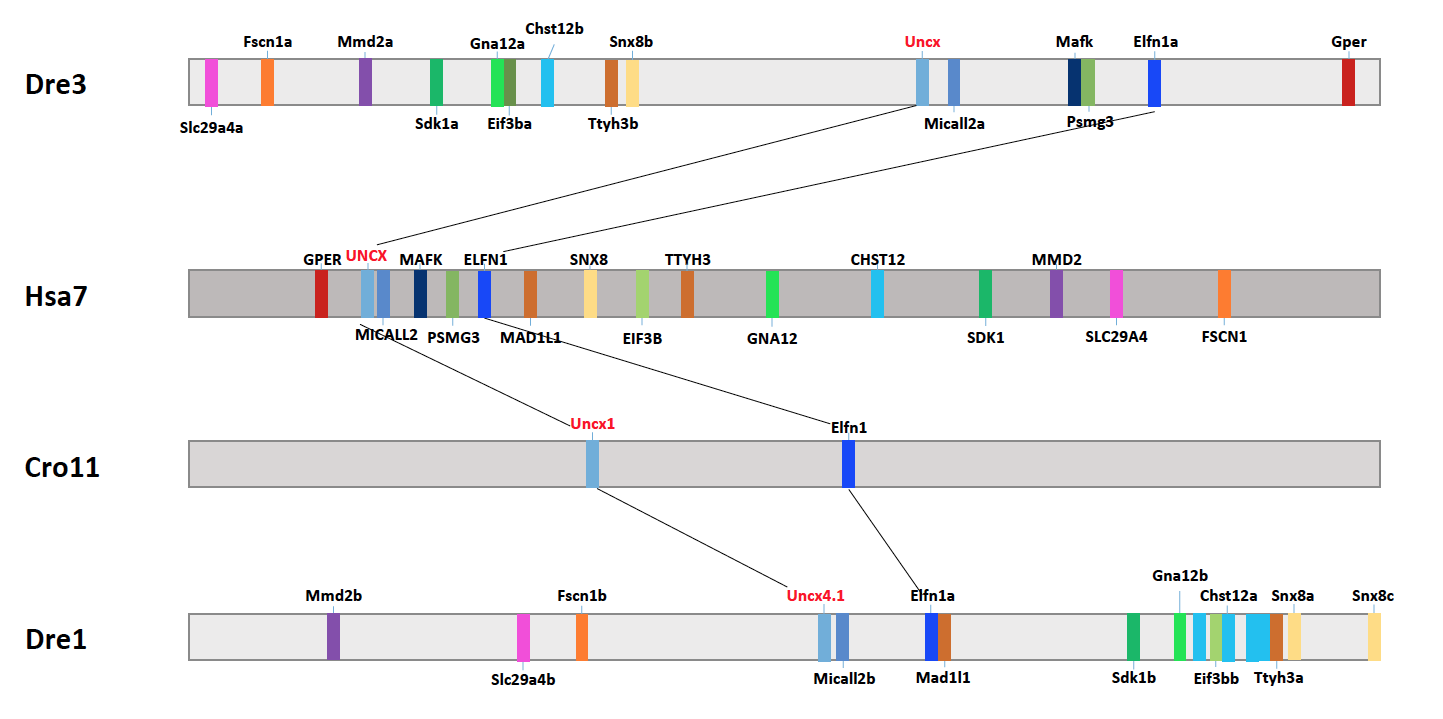


**Supplemental Figure 3**


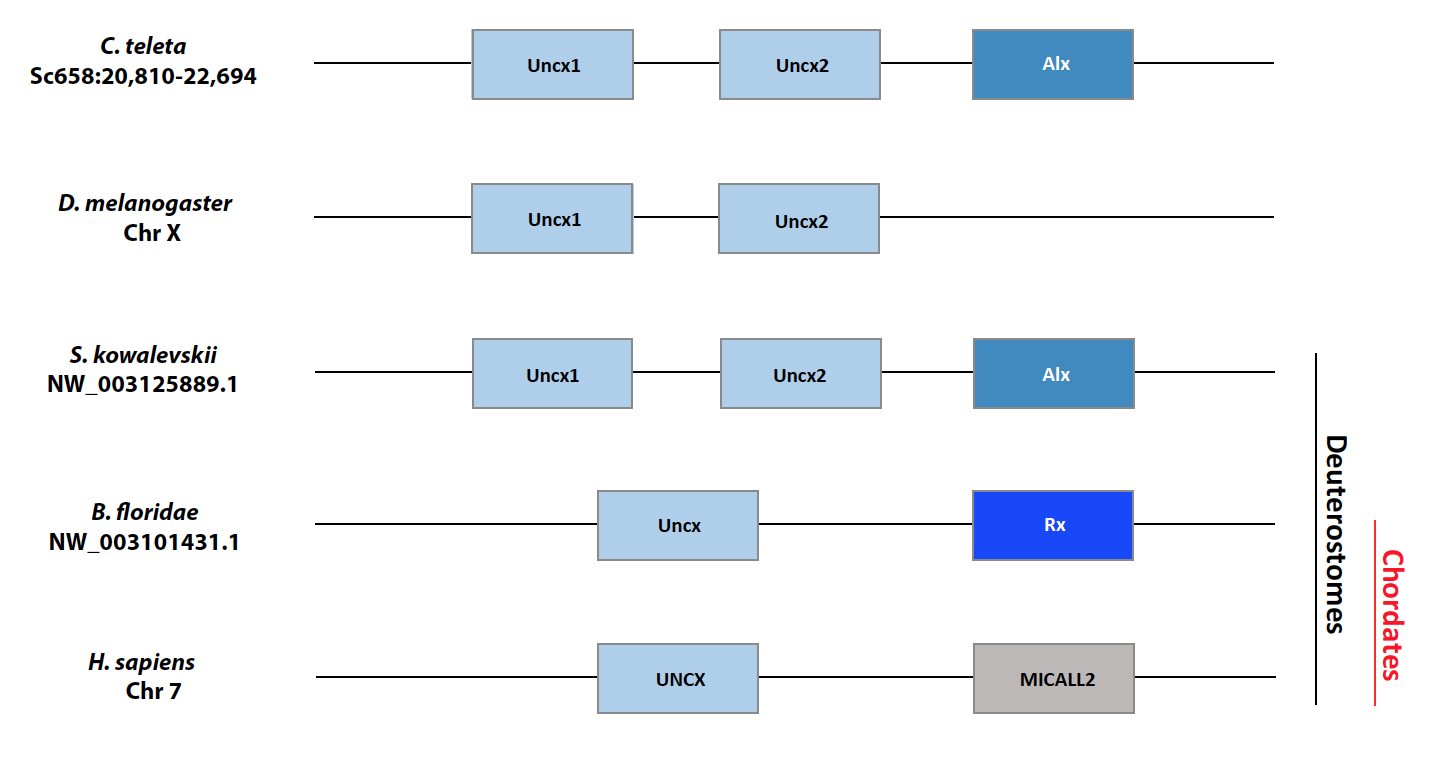


**Supplemental Figure 4**

**Supplemental Figure 5**

**
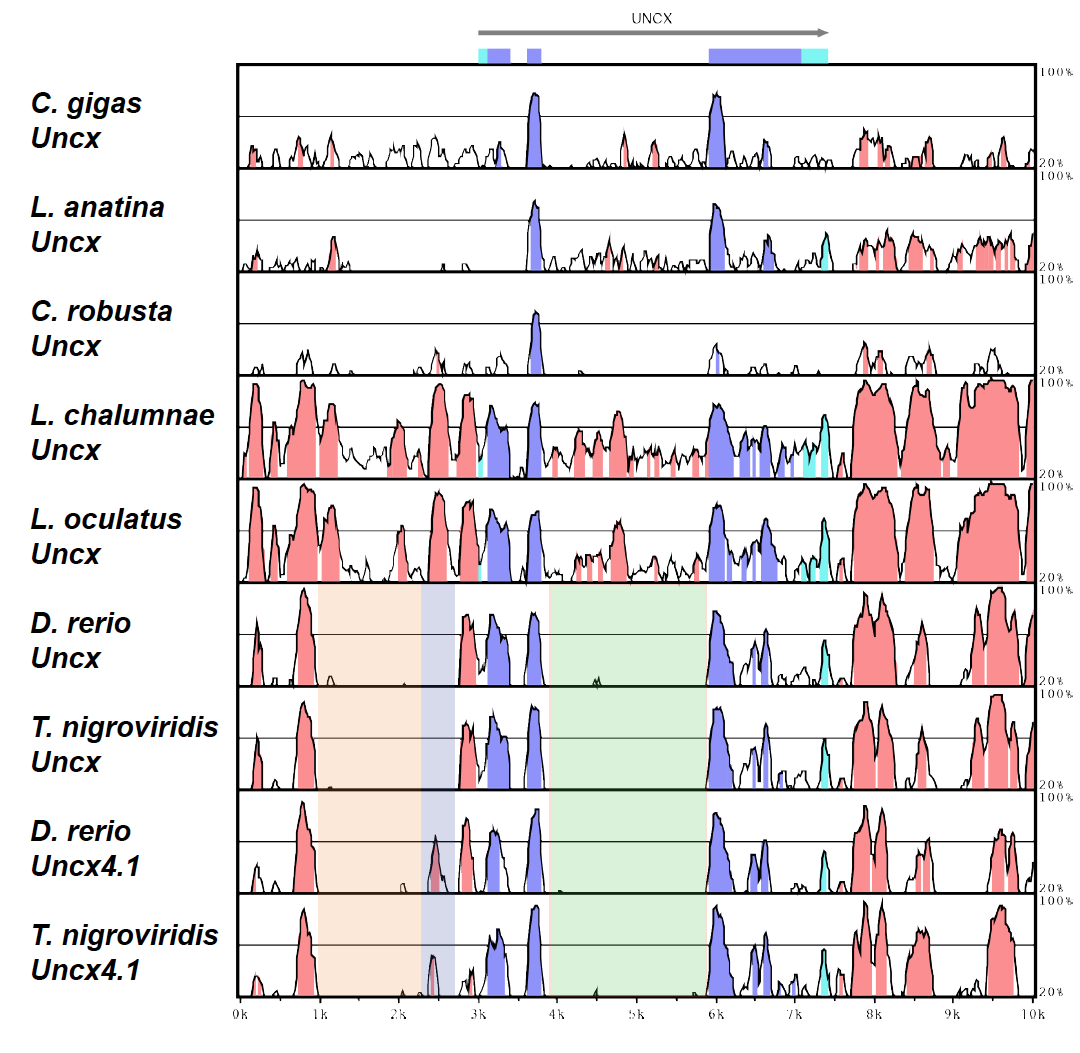
Supplemental Figure 6**

**
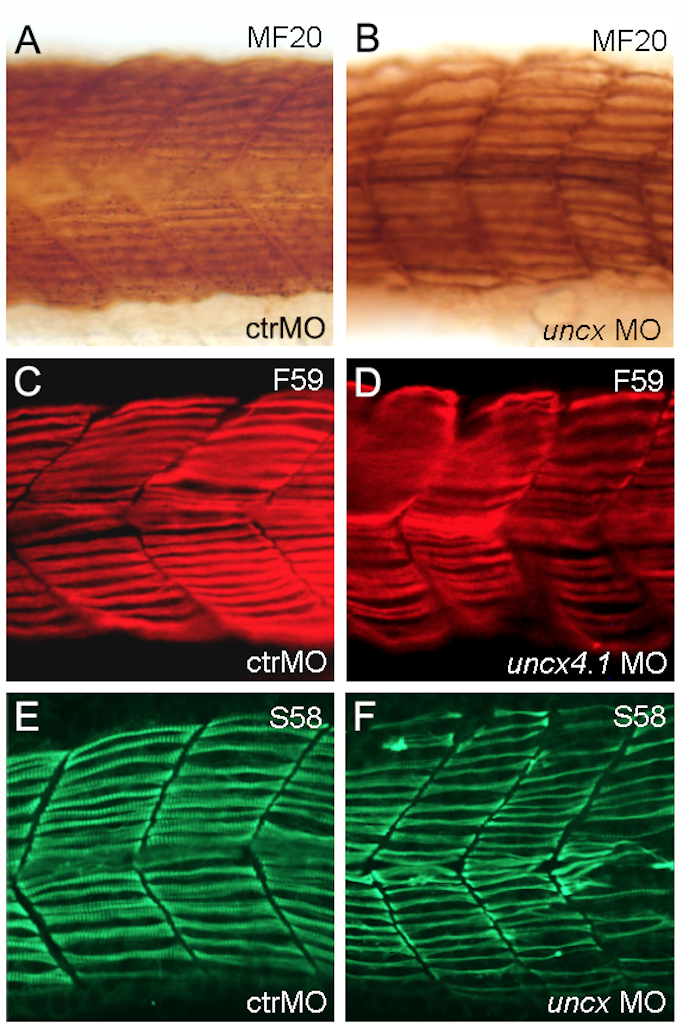
**

**Supplemental Figure 7**

**
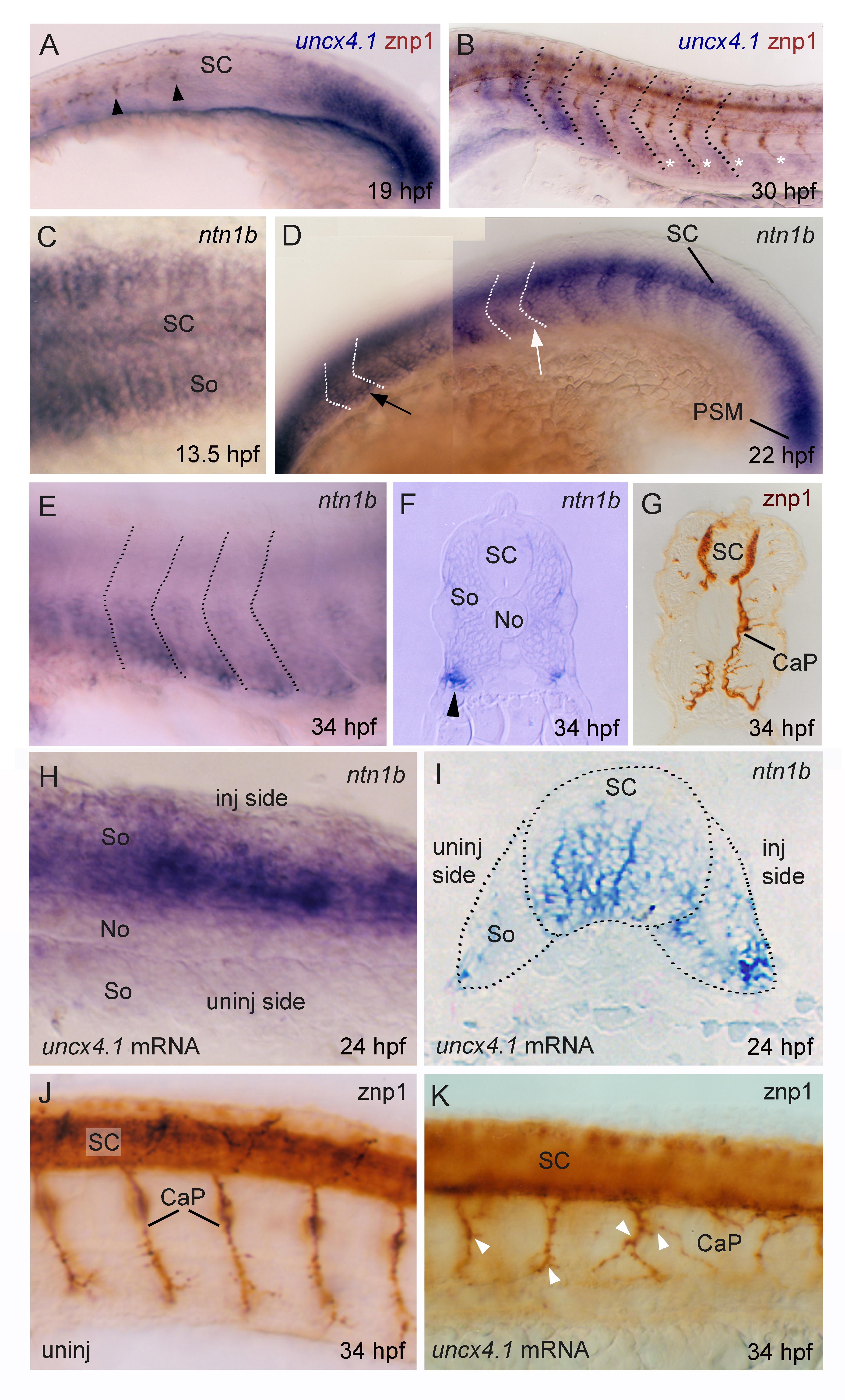
**
